# Disproportionation of Nitric Oxide at a Surface‐Bound Nickel Porphyrinoid

**DOI:** 10.1002/ange.202201916

**Published:** 2022-03-21

**Authors:** Matus Stredansky, Stefania Moro, Manuel Corva, Henning Sturmeit, Valentin Mischke, David Janas, Iulia Cojocariu, Matteo Jugovac, Albano Cossaro, Alberto Verdini, Luca Floreano, Zhijing Feng, Alessandro Sala, Giovanni Comelli, Andreas Windischbacher, Peter Puschnig, Chantal Hohner, Miroslav Kettner, Jörg Libuda, Mirko Cinchetti, Claus Michael Schneider, Vitaliy Feyer, Erik Vesselli, Giovanni Zamborlini

**Affiliations:** ^1^ Physics Department University of Trieste via A. Valerio 2 34127 Trieste Italy; ^2^ CNR-IOM, Area Science Park S.S. 14 km 163,5 34149 Trieste Italy; ^3^ Department of Physics TU Dortmund University Dortmund Germany; ^4^ Peter Grünberg Institute (PGI-6) Forschungszentrum Jülich GmbH Jülich Germany; ^5^ Department of Chemistry and Pharmaceutical Science University of Trieste via L-Giorgieri 1 34127 Trieste Italy; ^6^ Institut für Physik Karl-Franzens-Universität Graz 8010 Graz Austria; ^7^ Erlangen Center for Interface Research and Catalysis Friedrich-Alexander-Universität Erlangen-Nürnberg Egerlandstr. 3 91058 Erlangen Germany; ^8^ Fakultät f. Physik and Center for Nanointegration Duisburg-Essen (CENIDE) Universität Duisburg-Essen 47048 Duisburg Germany

**Keywords:** 2D Materials, Biomimetic Materials, Disproportionation, Nitrogen Monoxide, Porphyrins, Single-Atom Catalysts

## Abstract

Uncommon metal oxidation states in porphyrinoid cofactors are responsible for the activity of many enzymes. The F_430_ and P450nor co‐factors, with their reduced Ni^I^‐ and Fe^III^‐containing tetrapyrrolic cores, are prototypical examples of biological systems involved in methane formation and in the reduction of nitric oxide, respectively. Herein, using a comprehensive range of experimental and theoretical methods, we raise evidence that nickel tetraphenyl porphyrins deposited in vacuo on a copper surface are reactive towards nitric oxide disproportionation at room temperature. The interpretation of the measurements is far from being straightforward due to the high reactivity of the different nitrogen oxides species (eventually present in the residual gas background) and of the possible reaction intermediates. The picture is detailed in order to disentangle the challenging complexity of the system, where even a small fraction of contamination can change the scenario.

## Graphical abstract

Nickel tetraphenyl porphyrins deposited in vacuo on a copper surface are reactive towards nitric oxide disproportionation already at room temperature. The high chemical reactivity of nitrogen oxides species, eventually present in the residual gas background, and of the possible reaction intermediates yields a challenging complexity, where even a small fraction of contamination can change the scenario.

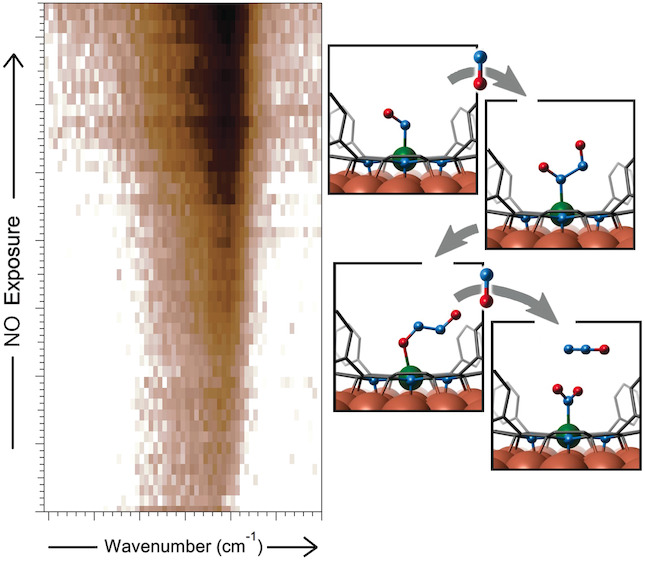

## Introduction

Enzymes are considered as a blueprint for novel synthetic catalysts that emulate their binding selectivity and high efficiency.^[1]^ The key constituents of several enzymatic reaction centers involved e.g. in methanogenesis,^[2]^ catalytic oxidation,^[3]^ and nitric‐oxide reduction^[4]^ are metal‐containing tetrapyrroles. Nitric oxide reductase (NOR) enzymes, such as NorBC and cytochrome P_450_, take also advantage of the selectivity provided by these single metal atom cores and by the electronic and geometric architecture surrounding the reactive site. One of the mechanisms proposed in the description of the NO conversion process in NOR involves, in the intermediate step, the formation of a hyponitrite (N_2_O_2_) moiety, resulting from the coupling of two NO molecules.^[5]^ This elusive species is hard to stabilize and detect directly.[^6^, ^7^] The production of nitrous oxide (N_2_O) by engineered myoglobins is found instead to proceed through a nitrosyl dimer (NO)_2_.^[8]^ More generally, when looking at the mechanistic aspects of the reactions of nitric oxide, either with transition metal (TM) complexes or at surfaces, it is observed that homomolecular reactions involve disproportionation mechanisms covering a variety of pathways and products.[^9^, ^10^] In the former case (TM complexes), the atom transfer processes observed at Ni, Cu, Fe, Mn, and Ru centers can involve 3 to 4 NO molecules, yielding N_2_O+NO_2_ or N_2_+2 NO_2_, respectively.^[9]^ Conversely, in the case of surfaces, atomic products can be easily accommodated, thus opening the way to additional parallel or competing paths. NO adsorption and reaction at Cu single crystal terminations in Ultra‐High Vacuum (UHV) yield adsorbed atomic O and N, together with N_2_O, NO_2_, NO_3_, and N_2_O_4_ already below 110 K.[^11^, ^12^, ^13^, ^14^] The origin of the complex chemistry of NO partly arises from its electronic structure, with an unpaired electron in its 2π* orbital, so that the molecule can act both as an electron donor or as an acceptor. However, this represents an oversimplified picture that does not account for many observations, e.g. reaction paths that involve NO dimers (NO)_2_ or hyponitrite intermediates (N_2_O_2_), making a thorough comprehension of this molecule's chemistry quite puzzling.^[7]^ Furthermore, experimental investigations involving NO as a reactant are challenging due to the presence of other N oxides as trace contaminants in the gas sources, thus making precise and clean measurements very complex, both in surface science and biochemistry.[^15^, ^16^] In the latter case, the first single‐site heterogeneous material to promote NO disproportionation into N_2_O and NO_2_ was reported only recently.^[17]^ The process was found to occur with low rate at Fe sites already at room temperature. Computational investigations of the observed 3 NO→N_2_O+NO_2_ reaction confirmed the role of a monoanionic hyponitrite radical intermediate in an exothermic pathway (3 eV) to gaseous N_2_O and adsorbed NO_2_ at the Fe site.[^17^, ^18^]

In a biomimetic picture at surfaces, novel model systems for heterogeneous catalysis may be built by exploiting tetrapyrrolic compounds,[^19^, ^20^] such as metal‐containing tetraphenyl porphyrins (MTPP). MTPP form a rich variety of 2‐dimensional self‐assembled structures when adsorbed at proper templating surfaces.^[21]^ These supramolecular assemblies act as a network, stabilizing ordered arrays of single metal atom active sites.[^22^, ^23^] In fact, within the macrocycle moiety of the porphyrin, the incorporated transition metal is considered a single‐atom catalyst (SAC),^[24]^ which offers axial coordination for the anchoring of ligands,[^25^, ^26^] and for the chemical conversion of small molecules.[^27^, ^28^, ^29^] In the specific case of NO adsorption at 2D arrays of surface‐supported porphyrins, it is commonly accepted that only a single NO molecule coordinates to the metal center under UHV conditions.[^25^, ^30^, ^31^, ^32^] In the present work, instead, we present the results obtained upon exposure of a Ni tetraphenyl porphyrin (NiTPP) film deposited on the Cu(100) surface to NO at room temperature, providing a first evidence of NO disproportionation observed at tetrapyrroles in UHV, yielding the NO_2_‐NiTPP complex. By means of vibronic and vibrational spectroscopies and scanning tunneling microscopy (STM), we unequivocally identify the ligand coordinated with the Ni atom of the porphyrin to be the NO_2_ molecule. Independent photoemission‐based experiments allow to draw the same conclusions. Density Functional Theory (DFT) calculations indicate that the disproportionation reaction path (3 NO→NO_2_+N_2_O) is extremely exothermic so that the formation of the NO_2_‐NiTPP complex is energetically favorable, although kinetically hindered, being a high order reaction. In the following we will report in detail the results obtained by means of each of the many techniques, both experimental and theoretical, that we exploited to investigate this complex system (see Supporting Information for further details about the adopted methodologies). We will also discuss our observations in light of the well‐known, unavoidable, and non‐trivial issues associated with the role of contaminants in NO experiments, critically considering the role of pathways including: i) NO_2_ contamination of the NO bottles, ii) NO_2_ formation at the UHV chamber walls, iii) NO_2_ formation at the Cu substrate, iv) NO_2_ formation at the single‐atom Ni sites.

## Results and Discussion

**Figure 1:**
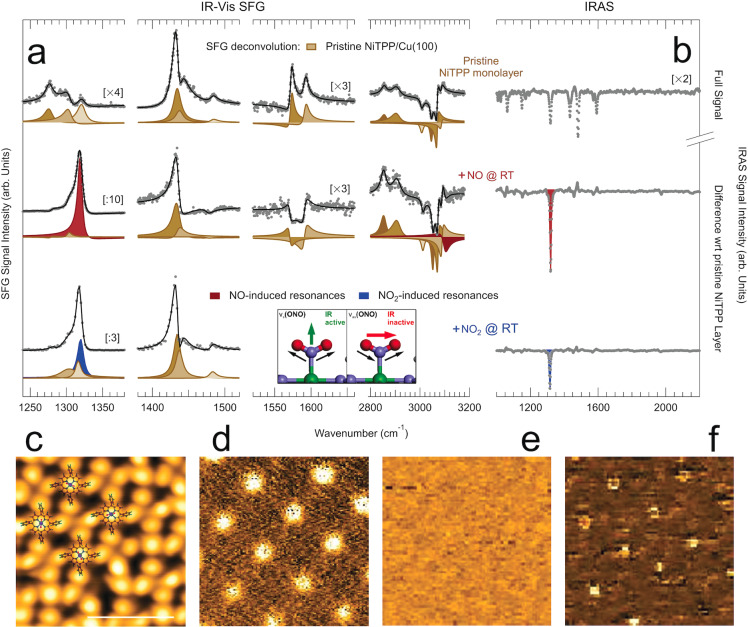
a) IR‐Vis SFG spectra of a pristine NiTPP/Cu(100) monolayer at room temperature before (top) and after exposure to NO (middle) and to NO_2_ (bottom). For the IR‐Vis SFG (IRAS) experiment the layer was exposed to 6×10^2^ L (5×10^2^ L) of NO and to 1 L (1.44 L) of NO_2_, respectively. Data are shown (grey markers) together with the best fit (black lines) and deconvolution (filled profiles) obtained according to the lineshape described in the Supporting Information. b) IRAS spectra corresponding to the IR‐Vis SFG spectra in (a). c) Constant‐height topographic STM image of the NiTPP/Cu(100) after saturation with NO with superimposed NiTPP models (bias +140 mV; bar corresponds to 2 nm). d–f) Inelastic electron tunneling d^2^I/dV^2^ maps at constant height of the same zone in (c) collected at 20, 140, and 170 mV bias, respectively.

It has been demonstrated that the charge transfer taking place at the NiTPP/Cu(100) interface yields population of the molecular orbitals up to the LUMO+3,^[33]^ strongly affecting the metal oxidation state.^[34]^ This results in a unique electronic configuration of the single metal atom site with potentially high reactivity,^[35]^ associated with thermal stabilization^[36]^ and a strong surface trans‐effect.^[37]^ NiTPP molecules self‐assemble on the Cu(100) surface at monolayer coverage forming two long‐range ordered domains that are commensurate with the underlying substrate.^[33]^ Infrared‐Visible Sum‐Frequency Generation (IR‐Vis SFG) and Infrared Reflection Absorption Spectroscopy (IRAS) spectra reveal the presence of several resonances (Figure 1a, b, top), associated with the vibrational modes of the different porphyrin moieties (Table 1),[^38^, ^39^, ^40^] in agreement with previous observations.^[28]^ When exposing the system to NO, a sharp and intense resonance grows in the IR‐Vis SFG spectra at 1319 cm^−1^ (Figure 1a, middle, red envelope), evolving both in amplitude and phase with NO exposure (10^1^–10^3^ L) and showing a lineshape dependence on the initial NiTPP surface coverage (Figures S1, S2, Tables S1–S5). IRAS spectra confirm the growth of a strong absorption feature at the same energy (Figure 1b, middle, red envelope), indicating a strong dipole mode with a significant component in the direction orthogonal to the surface plane. However, the stretching contribution of terminal NO would be expected above 1700 cm^−1^.^[41]^ Comparison with literature data leads instead to an unexpected conclusion,^[42]^ associating the vibration at 1319 cm^−1^ with the asymmetric stretch of an adsorbed NO_2_ species. As a counter experiment, we expose the pristine NiTPP monolayer at room temperature directly to 1 L of NO_2_. The growth of the same features is readily observed both in IR‐Vis SFG and IRAS experiments (Figure 1a, b, bottom, cyan envelopes, and Figure S3). By comparing the STM images acquired before and after the NO uptake, we note that the dark depression at the macrocycle center, which is associated with the Ni atom,[^33^, ^35^] is replaced with a bright protrusion (Figure 1c), similarly to what has been observed upon direct exposure to NO_2_ (Figure S4).^[35]^ The NO_2_ ligand coordinates to the Ni atom at the center of the macrocycle, as further confirmed by the STM d^2^I/dV^2^ maps. Inelastic Electron Tunneling Spectroscopy localizes indeed the mode low‐frequency mode (Figure 1d) and the mode detected by means of both IR‐Vis SFG and IRAS (Figure 1f) on top of the Ni atoms, whereas no vibrational feature was localized at intermediate bias (Figure 1e). The scenario depicted above is confirmed by X‐ray Photoemission Spectroscopy (XPS) (Figure S5). The main Ni 2*p*_3/2_ peak at a binding energy of 852.9±0.3 eV, assigned to the core level of the central Ni ion in a formal +1 oxidation state of the pristine system,[^34^, ^35^] is progressively replaced by a new feature at higher binding energy (854.6±0.3 eV) after exposure of the NiTPP film to NO or, equivalently, to NO_2_. The observed binding energy shift witnesses the NO_2_‐induced oxidation of the nickel atom, confirming ligation at the single metal atom site. Consistently, while the N 1s spectrum of the pristine NiTPP‐covered surface shows a single, sharp peak at 398.65±0.2 eV, assigned to the four equivalent nitrogen atoms of the macrocycle,[^43^, ^44^] a new spectral feature adds at 402.55±0.2 eV upon exposure of the molecular film to NO or NO_2_, associated with the N atom of the NO_2_ ligand.^[35]^ As both vibrational and electronic properties are unique for the specific molecule, all experimental techniques therefore point towards the adsorption of NO_2_ at the NiTPP/Cu(100) layer, even after exposure to NO.

**Table 1:** Deconvolution parameters and assignment, according to the literature,[^38^, ^39^, ^40^] of the IR‐Vis SFG resonances observed for the pristine monolayer of NiTPP/Cu(100) and of observed IRAS absorption lines for the same system (see Supporting Information for further data and details); in the bottom part of the table, IR‐Vis SFG and IRAS features specifically induced by the reaction with NO are reported.^[42]^

This work			Literature		Literature	
IRAS	IR‐Vis SFG		Phenyl Modes		Macrocycle Modes	
*ω* [cm^−1^]	*ω* [cm^−1^]	Δ*ϕ* [°]	*ω* [cm^−1^]	Assignment	*ω* [cm^−1^]	Assignment
1063			1050–1071	in‐plane, out‐of‐plane		δ(C_β_‐H)_sym_
1150			1152–1158	out‐of‐plane		
1172			1177	in‐plane	1190	δ(C_β_‐H)_asym_
	1276–1279	288	1269	δ(CH)	1269	ν(C_m_‐Ph), ν(NC_α_)
	1304–1305	350			1302	ν(pyr half‐ring), ν(NC_α_), ν(C_α_C_β_)
1319	1315–1319	224	1317, 1318	out‐of‐plane B_1u_, A_2u_	1313	ν(pyr quarter‐ring)
1434	1434	246	1438	out‐of‐plane B_1u_		
	1436–1437	97	1440	out‐of‐plane A_2u_		
1481	1482–1483	215	1470	δ(CCH), ν(CC)	1470, 1473, 1485	ν(C_α_C_m_)_sym_, ν(C_β_C_β_), ν(NC_α_), ν(C_α_C_β_)
1571	1573	105	1576, 1583, 1586	ν(CC), out‐of‐plane E_g_, B_1u_, A_2u_	1572	ν(C_β_C_β_), ν(C_α_C_m_), δ(C_α_C_m_)
1592	1593	238	1586	out‐of‐plane A_2u_	1586, 1594	ν(C_α_C_m_)_sym_, ν(C_α_C_m_)_asym_, δ(C_α_C_m_Ph)
	2856	280				
	2907	295				
	3007	87	3039, 3047, 3063, 3068, 3069, 3071, 3073, 3075	ν(CH), out‐of‐plane E_g_, B_1u_, A_2u_		
3047	3046	97				
3069	3069	131				
	3086	155				
				
This work—NO Uptake			Literature			
IRAS	IR‐Vis SFG					
*ω* [cm^−1^]	*ω* [cm^−1^]	Δ*ϕ* [°]	*ω* [cm^−1^]	Assignment		
1319	1319	7	1304–1311	NO_2_ asymm. Stretch		
	1365	250	1374, 1377	(only above ≈10^3^ L NO) ν(pyr quarter/half‐ring)		
	1602	215	1594, 1599	(only above ≈10^3^ L NO) ν(C_α_C_m_)_asym_, in‐plane phenyl		
	3097–3101	45	3095–3100	ν(CH) in‐plane		

In the case of larger exposures to NO, (>10^3^ L), the IR‐Vis SFG resonance lineshape at 1319 cm^−1^ considerably evolves due to a phase rotation (Figure S2 and Tables S1–S3). The latter is accompanied by the appearance of additional resonances associated with the NiTPP molecules at 1365, 1602, and at about 3100 cm^−1^. This behavior is explained by a distortion of the macrocycle and phenyl moieties, yielding a change in the orientation of the dipole moments pertaining to the corresponding vibrational modes (see Table 1). The relative phase rotation of the spectroscopic resonances with respect to the non‐resonant background reflects a change of the density of states in an energy range around the Fermi level that is compatible with the visible photon energy (532 nm, 2.33 eV) exploited in the measurements.[^45^, ^46^, ^47^] The modifications induced in the IR‐Vis SFG NiTPP resonances by large doses of NO are in line with what previously observed for adsorption of NiTPPs directly on an oxygen pre‐covered Cu(100) termination, associated with a decoupling effect induced by the underlying (2√2×√2)R45°‐O/Cu(100) superstructure.^[28]^ Thus, we observe the parallel, progressive oxidation of the supporting Cu(100) surface occurring with prolonged exposures to NO (of the order of 10^3^ L) of the NiTPP/Cu(100) system. XPS measurements of the O 1s core level (Figures S5, S6) agree with the oxygen‐induced passivation of the copper surface. Indeed, whereas it is straightforward that the as‐deposited NiTPP film does not show any trace of oxygen, after exposing the molecular layer to NO (NO_2_), two components grow at 531.1 and 529.7±0.2 (530.1±0.2) eV, with very different relative intensities. By comparing the data with the exposure of the bare Cu(100) surface, on which NO is known to dissociate readily (Figure S6), we conclude that the high binding energy feature is associated with the NO_2_ ligand at the Ni sites, while the low energy peak is assigned to atomic O at the copper surface, with binding energy shifts depending on the O coverage.[^12^, ^48^, ^49^] The actual intensity ratio of the two O 1s components—NO_2_/NiTPP vs. O/Cu(100)—is ultimately determined by i) the initial NiTPP coverage, eventually leaving bare Cu(100) islands when less than a monolayer is deposited, and ii) by the degree of oxidation of the Cu substrate that is reached upon prolonged exposures, in perfect agreement with the IR‐Vis SFG and IRAS (Figure S7) results. Accordingly, we exclude formation of NO_2_ at the bare Cu(100) termination.

**Figure 2:**
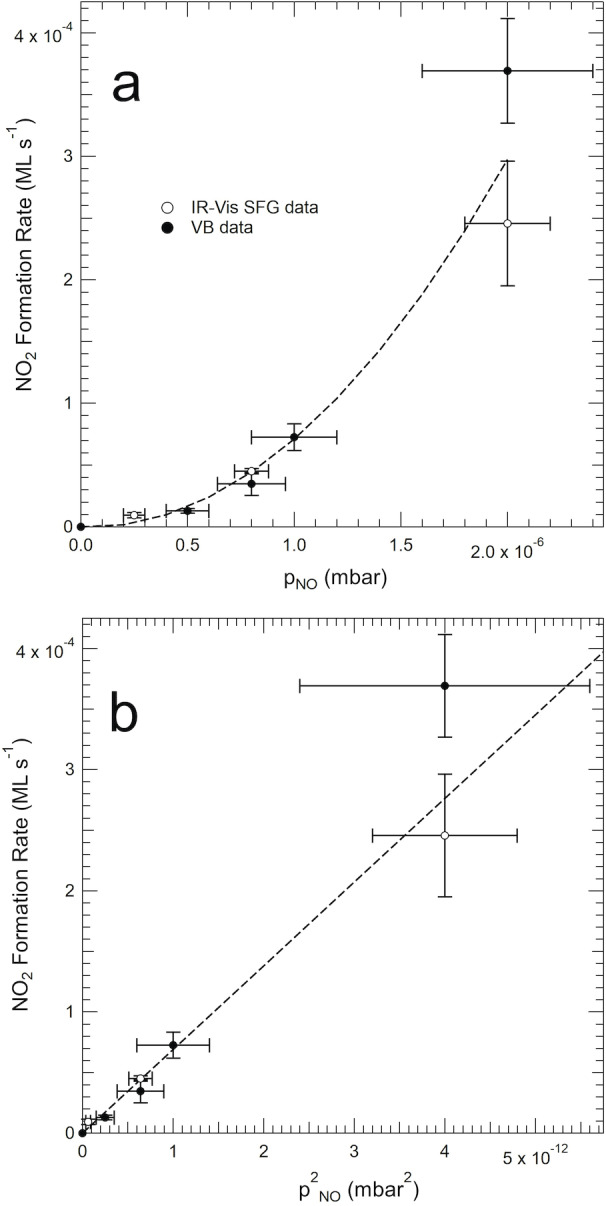
Combined IR‐Vis SFG (empty markers) and VB (filled markers) data of the NO uptakes on a NiTPP/Cu(100) monolayer at RT, showing the NO_2_/NiTPP formation rate as a function of the NO uptake background pressure (a) and pressure squared (b).

As pinpointed in the introduction, the chemistry of nitrogen oxides deserves particular attention due to the high reactivity of these molecules, in association with the possible presence/formation of contaminants during the experiment. The IR‐Vis SFG, IRAS, STM, and XPS measurements presented here were performed in different setups (see Methods), using different NO bottles and with different NO purification and handling recipes, nevertheless always yielding the same conclusions, i.e. the formation of NO_2_ at the Ni sites after exposure of the NiTPP/Cu(100) layer to NO at room temperature. Concerning the results presented here, the remaining alternative interpretation pathways are: i) since there is no way to get rid of NO_2_ contamination, always present in NO bottles, and considering the extremely NO_2_ high sticking coefficient, impurities of the order of 0.1 % or even less could give rise to a signal intensity (XPS, VB, IR‐Vis SFG, IRAS) or STM features compatible with the observed ones; ii) NO reacts at the internal walls of the UHV setups, generating a whole family of nitrogen oxides,[^9^, ^10^, ^12^, ^13^, ^15^, ^50^] including NO_2_ that ultimately sticks at the sample's surface; iii) NO disproportionation effectively occurs at the Ni sites of the single‐atom biomimetic catalyst layer. Despite the large set of experimental techniques and approaches adopted up to here, yet it was not possible to discriminate among the possible different pathways. Thus, to address this issue, we designed a combined experiment that was performed independently in two different experimental setups by means of time‐dependent IR‐Vis SFG and Valence Band (VB) measurements, respectively. In particular, the evolution of the NiTPP/Cu(100) monolayer was followed as a function of time during a series of NO uptakes performed at room temperature and different (constant) pressure values. IR‐Vis SFG spectra were collected in situ in NO background in the 1280–1340 cm^−1^ range (Figure S8a) to monitor the evolution of the resonance at 1319 cm^−1^, associated with the formation/adsorption of NO_2_. Several uptakes were performed at different NO background pressure values (Figure S8b) and the initial NO_2_ formation rate was measured. Similarly, but independently, the same information was obtained by stepwise exposures to NO alternating with VB measurements in UHV (Figures S9, S10). The combined results (empty and filled markers) reported in Figure 2 both as a function of p_NO_ and p_NO_^2^, reveal that the NO uptake rate is non‐linear as a function of the reactant pressure, so that the NO_2_ formation rate is proportional to the square of p_NO_. This supports a disproportionation process, in the direction of excluding a possible role of contaminants in the gas sources.

**Figure 3:**
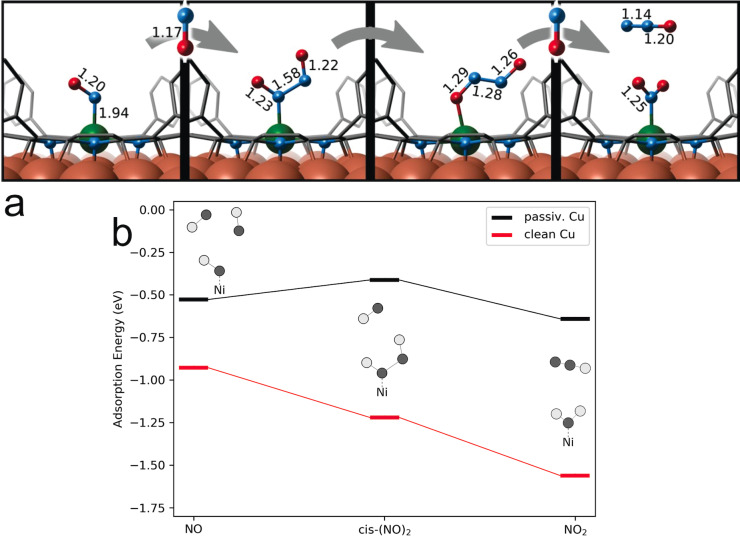
a) Reaction pathway for NO disproportionation catalyzed by NiTPP/Cu(100) as suggested by DFT calculations: bond lengths obtained from the lowest energy configurations are indicated (see Table 2 for further details). Atoms color coding: oxygen (red), nitrogen (cyan), nickel (green), copper (orange). b) Calculated adsorption energies (eV—PBE‐D3/VASP) of NO_*x*_ species to NiTPP on the clean and passivated Cu surface.

So far, we have narrowed our potential NO_2_ source down disproportionation reactions at the UHV chamber walls or at our Ni‐catalyst layer. At this point, further corroboration comes from theory. We performed a thorough set of ab initio calculations within the framework of Density Functional Theory (DFT) to shed light on the proposed NO disproportionation mechanism (Figure 3 and Tables S6–S8. Following pathways similar to those suggested for NO conversion in metal‐organic biochemical systems,[^17^, ^51^, ^52^, ^53^, ^54^] our set includes different NO_*x*_ intermediates along the stepwise addition of NO to the Ni site (Figure 3a). Of course, not all intermediates are expected to be observable and to yield a sizeable surface coverage, depending on the configuration lifetime in relation with reaction barriers and other kinetic parameters (pre‐exponentials, coverage, reactant pressure and temperature, surface temperature…). After the adsorption of the first NO molecule, we assume formation of the (NO)_2_ dimer,[^55^, ^56^, ^57^, ^58^] which is firstly converted into hyponitrite and then to NO_2_ (and N_2_O) consuming a third NO. We start by discussing the influence of both the NiTPP complex and the Cu‐surface on such a pathway and link the observations to the changes induced in the ligated NO_*x*_ species. Table 2 compares the bond lengths of NO, (NO)_2_, and NO_2_ in different environments. In absence of the surface trans‐effect, i.e. without the Cu surface below the porphyrin, the ligands are negligibly influenced, nor geometrically distorted by the NiTPP molecule (Table 2, rows 1 and 2). Accordingly, the local magnetic moments and projected density of states resemble the isolated NO_*x*_ and NiTPP species (Table S6). This behavior is preserved also when including an oxygen reconstructed Cu‐surface in the simulations (3^rd^ row of Table 2), which in turn underlines the capability of decoupling porphyrin and surface by oxygen passivation, as recently observed.[^27^, ^28^] On the metallic Cu(100) surface, however, the situation is fundamentally different due to the strong *trans*‐effect, associated with charge transfer from the substrate to the metal–organic complex, with the Ni atom changing from a formally *d* ^8^ to a *d* ^9^ configuration. The increased electron density at the Ni center allows for electron donation from the Ni *d*‐orbital to an empty π*‐antibonding orbital of the NO_*x*_ ligand. Thus, in the simulations, the NO_*x*_ ligands are better described as their anionic counterparts. As common for such a π‐back‐bonding, the Ni−NO_*x*_ bond is strengthened, while the ligand's NiN−O_*x*_ bonds are weakened compared to the gas phase (or to the oxygen‐passivated) case. In terms of bond lengths, this is reflected in a severe decrease of the Ni−NO_*x*_ distance from 2.2 to 2.0 Å, accompanied by an increased NiN−O_*x*_ bond length for all species. NO is found to coordinate in a bent conformation, which is often seen as characteristic for an increased anionic character of the ligand in metal‐nitrosyl complexes.^[32]^ Note that the fundamental role of Cu(100) in enabling charge donation towards a Ni *d* ^9^ configuration is further confirmed by simulations of the charged NO_*x*_/NiTPP complex in the gas phase, yielding similar geometric distortions when compared to the neutral counterpart. The most intriguing effect of back‐bonding, however, can be observed for the adsorption of the (NO)_2_ dimer. In the gas phase, the weak interaction between the two NO moieties results in an unusually long N−N distance of 2 Å. This weak bonding in the dimer remains unchanged upon interaction with a Ni *d* ^8^ center. However, upon adding an additional electron to the metal, thus taking into account the charge transfer induced by the metallic Cu surface, the N−N distance decreases to 1.6 Å, stabilizing a monoanionic hyponitrite (N_2_O_2_^−^) species.[^59^, ^60^] While formation of (NO)_2_ is hardly observed at ambient conditions, the stabilization of a hyponitrite intermediate is a reasonable first step in the NO disproportionation reaction. Note that (NO)_2_ as well as N_2_O_2_ may be present in their *cis*‐ or *trans*‐configurations with the corresponding isomers close in energy. The already bent coordination of the first NO at the Ni center may initially facilitate the adsorption of the second NO in a *cis*‐configuration. However, we cannot explicitly conclude the occurrence of a (short‐lived) di‐nitrosyl species at NiTPP, making a clear distinction between Langmuir–Hinshelwood and Eley–Rideal reaction mechanisms hard to address. A possible pathway could then include the isomerization to the *trans*‐form and reaction with a third NO, similarly to what proposed for other systems.^[61]^ The energetic consequence of the π‐back‐bonding capability of the active NiTPP/Cu(100) phase (at variance with the NiTPP/O/Cu(100) case) is the increasingly favorable adsorption of each NO_*x*_ species along the reaction coordinate (Figure 3b and Table S7). The calculated NO_*x*_ adsorption energies point toward an increased fixation of each species at the Ni center, which in turn facilitates the addition of the next NO. Independently, inferred from the relative electronic energies, the thermodynamic equilibrium of the reaction 3 NO→NO_2_+N_2_O favors NO disproportionation (compare lines in Table S8), although in the gas phase this reaction is kinetically hindered.^[62]^ A full mechanistic study is, however, beyond the scope of this work, especially as many intermediates are expected to be short‐lived, hard to access, and thus difficult to detect experimentally. Regardless of the exact pathway, NO_2_ is anyway formed along the way together with possible side products such as N_2_O or N_2_. In contrast to NO_2_, those side products, however, do not interact with the Ni *d* ^9^ center, with equilibrium N−Ni distances beyond 3 Å in our calculations. While the calculations do not specifically exclude the formation of NO_2_ at the chamber walls, the show the increasing stabilization of possible disproportionation intermediates at the NiTPP due to the unique electronic configuration of the Ni center. Moreover, our calculations support the experimental finding of stable NO_2_ as a result of NO conversion, while other reaction products go undetected.

**Table 2:** Bond lengths (Å) of gas phase and adsorbed NO_*x*_ and (NO_*x*_)‐NiTPP species calculated with PBE+D3/VASP. In the coordinated hyponitrite species, the two N−O bonds are not equivalent anymore: Here (*), the N−O distance is given for the Ni‐coordinated N, while the second N−O bond is 1.22 Å.

	NO	*cis*‐(NO)_2_			NO_2_		
	N−O	N−Ni	N−O	N−N	N−Ni	N−O	N−Ni
NOx	1.17	–	1.18	2.00	–	1.21	–
NOx‐NiTPP	1.17	2.17	1.19	1.96	2.40	1.23	2.23
NOx‐NiTPP/O/Cu(100)	1.18	2.21	1.18	1.98	2.26	1.24	2.21
NOx‐NiTPP/Cu(100)	1.20	1.94	1.23 (*)	1.58	1.99	1.25	2.00

## Conclusion

We have reported experimental evidence, obtained by exploiting several, independent approaches, that a stable NO_2_ species forms at the Ni sites at room temperature upon exposure of a NiTPP/Cu(100) monolayer to NO. The presence and stability of the ligand is solid and was proven by means of a counter‐experiment, where NO_2_ was directly dosed. However, we analyzed in detail the NO_2_ formation pathway by considering all possible routes, starting from contribution of the Cu(100) substrate, gas contamination, reactivity of the walls of the experimental setups, and ending by considering the effective catalytic activity of the SAC Ni sites. The NO uptake was characterized by a quantitative analysis of the NO_2_ formation rates as a function of the NO exposure conditions. As a consequence, we could disentangle the mechanism of NO_2_ formation from any interpretation due to possible NO_2_ residual contamination, putting in evidence the tough challenges related with nitrogen oxides and their reaction products associated with the misinterpretation of experimental results. We conclude that NO_2_ origins through a disproportionation mechanism. Nevertheless, despite the adoption of many gimmicks (pre‐conditioning of the UHV setup, gas sniffer, different gas line materials, lines flushing, traps, different pressure gauges…), still, a contribution from the chamber walls could not be ultimately ruled out. Together with the support of ab initio simulations, we propose an atomistic model of the reaction that is compatible with our observations. It is based on the initial step of coordination of one nitric oxide molecule to the Ni^I^ reactive site, followed by the intermediate capture of two additional NO molecules, and by the final release of N_2_O, which leaves a stable NO_2_ molecule at the Ni site. This model paves the way towards further investigations on NO disproportionation at biomimetic single‐atom sites within the framework of surface science.

## Conflict of interest

The authors declare no conflict of interest.

1

## Supporting information

As a service to our authors and readers, this journal provides supporting information supplied by the authors. Such materials are peer reviewed and may be re‐organized for online delivery, but are not copy‐edited or typeset. Technical support issues arising from supporting information (other than missing files) should be addressed to the authors.

Supporting Information

## Data Availability

The data that support the findings of this study are available from the corresponding author upon reasonable request.

## References

[1] R. J. Hooley , Nat. Chem. 2016, 8, 202–204. 26892547 10.1038/nchem.2461

[2] S. J. Moore , S. T. Sowa , C. Schuchardt , E. Deery , A. D. Lawrence , J. V. Ramos , S. Billig , C. Birkemeyer , P. T. Chivers , M. J. Howard , S. E. J. Rigby , G. Layer , M. J. Warren , Nature 2017, 543, 78–82. 28225763 10.1038/nature21427 PMC5337119

[3] B. Meunier , Chem. Rev. 1992, 92, 1411–1456.

[4] T. Hino, Y. Matsumoto, S. Nagano, H. Sugimoto, Y. Fukumori, T. Murata, S. Iwata, Y. Shiro, *Science* **2010**, *330*, 1666–1670. 10.1126/science.1195591 21109633

[5] A. M. Wright , T. W. Hayton , Inorg. Chem. 2015, 54, 9330–9341. 25928662 10.1021/acs.inorgchem.5b00516

[6] W. A. Brown , P. Gardner , D. A. King , J. Phys. Chem. 1995, 99, 7065–7074.

[7] W. A. Brown , D. A. King , J. Phys. Chem. B 2000, 104, 2578–2595.

[8] H. Matsumura , T. Hayashi , S. Chakraborty , Y. Lu , P. Moënne-Loccoz , J. Am. Chem. Soc. 2014, 136, 2420–2431. 24432820 10.1021/ja410542z PMC4004238

[9] P. C. Ford , I. M. Lorkovic , Chem. Rev. 2002, 102, 993–1018. 11942785 10.1021/cr0000271

[10] A. Shiotari , H. Koshida , H. Okuyama , Surf. Sci. Rep. 2021, 76, 100500.

[11] C. M. Kim , C.-W. Yi , D. W. Goodman , J. Phys. Chem. B 2002, 106, 7065–7068.

[12] D. W. Johnson , M. H. Matloob , M. W. Roberts , J. Chem. Soc. Faraday Trans. 1 1979, 75, 2143.

[13] A. T. Wee , J. Lin , A. C. Huan , F. Loh , K. Tan , Surf. Sci. 1994, 304, 145–158.

[14] J. F. Wendelken , Appl. Surf. Sci. 1982, 11–12, 172–185.

[15] J. Wang , B. E. Koel , J. Phys. Chem. A 1998, 102, 8573–8579.

[16] I. M. Lorković , P. C. Ford , Inorg. Chem. 2000, 39, 632–633. 11272555 10.1021/ic9910413

[17] C. K. Brozek , J. T. Miller , S. A. Stoian , M. Dincă , J. Am. Chem. Soc. 2015, 137, 7495–7501. 25988850 10.1021/jacs.5b03761

[18] J. Jover , C. K. Brozek , M. Dincǎ , N. López , Chem. Mater. 2019, 31, 8875–8885.

[19] E. Vesselli , J. Phys. Mater. 2020, 3, 022002.

[20] E. Vesselli , Nanoscale Adv. 2021, 3, 1319–1330. 36132852 10.1039/d0na00827c PMC9417665

[21] J. M. Gottfried , Surf. Sci. Rep. 2015, 70, 259–379.

[22] B. Hulsken , R. Van Hameren , J. W. Gerritsen , T. Khoury , P. Thordarson , M. J. Crossley , A. E. Rowan , R. J. M. Nolte , J. A. A. W. Elemans , S. Speller , Nat. Nanotechnol. 2007, 2, 285–289. 18654285 10.1038/nnano.2007.106

[23] K. S. Mali , N. Pearce , S. De Feyter , N. R. Champness , Chem. Soc. Rev. 2017, 46, 2520–2542. 28401976 10.1039/c7cs00113d

[24] X. Yang , A. Wang , B. Qiao , J. Li , J. Liu , T. Zhang , Acc. Chem. Res. 2013, 46, 1740–1748. 23815772 10.1021/ar300361m

[25] W. Hieringer , K. Flechtner , A. Kretschmann , K. Seufert , W. Auwärter , J. V. Barth , A. Görling , H.-P. Steinrück , J. M. Gottfried , J. Am. Chem. Soc. 2011, 133, 6206–6222. 21462965 10.1021/ja1093502

[26] C. Wäckerlin , K. Tarafder , J. Girovsky , J. Nowakowski , T. Hählen , A. Shchyrba , D. Siewert , A. Kleibert , F. Nolting , P. M. Oppeneer , T. A. Jung , N. Ballav , Angew. Chem. Int. Ed. 2013, 52, 4568–4571; 10.1002/anie.201208028 23512489

[27] I. Cojocariu , H. M. Sturmeit , G. Zamborlini , A. Cossaro , A. Verdini , L. Floreano , E. D'Incecco , M. Stredansky , E. Vesselli , M. Jugovac , M. Cinchetti , V. Feyer , C. M. Schneider , Appl. Surf. Sci. 2020, 504, 144343.

[28] M. Stredansky , S. Moro , M. Corva , M. Jugovac , G. Zamborlini , V. Feyer , C. M. Schneider , I. Cojocariu , H. M. Sturmeit , M. Cinchetti , A. Verdini , A. Cossaro , L. Floreano , E. Vesselli , J. Phys. Chem. C 2020, 124, 6297–6303.

[29] F. Sedona , M. Di Marino , D. Forrer , A. Vittadini , M. Casarin , A. Cossaro , L. Floreano , A. Verdini , M. Sambi , Nat. Mater. 2012, 11, 970–977. 23085570 10.1038/nmat3453

[30] K. Seufert , W. Auwärter , J. V. Barth , J. Am. Chem. Soc. 2010, 132, 18141–18146. 21126085 10.1021/ja1054884

[31] C. Wäckerlin , D. Chylarecka , A. Kleibert , K. Müller , C. Iacovita , F. Nolting , T. A. Jung , N. Ballav , Nat. Commun. 2010, 1, 61. 20975713 10.1038/ncomms1057 PMC2964454

[32] C. Isvoranu , B. Wang , E. Ataman , J. Knudsen , K. Schulte , J. N. Andersen , M.-L. L. Bocquet , J. Schnadt , J. Phys. Chem. C 2011, 115, 24718–24727.

[33] G. Zamborlini , D. Lüftner , Z. Feng , B. Kollmann , P. Puschnig , C. Dri , M. Panighel , G. Di Santo , A. Goldoni , G. Comelli , M. Jugovac , V. Feyer , C. M. Schneider , Nat. Commun. 2017, 8, 335. 28839127 10.1038/s41467-017-00402-0 PMC5570996

[34] G. Zamborlini , M. Jugovac , A. Cossaro , A. Verdini , L. Floreano , D. Lüftner , P. Puschnig , V. Feyer , C. M. Schneider , Chem. Commun. 2018, 54, 13423–13426. 10.1039/c8cc06739b 30427327

[35] H. M. Sturmeit , I. Cojocariu , A. Windischbacher , P. Puschnig , C. Piamonteze , M. Jugovac , A. Sala , C. Africh , G. Comelli , A. Cossaro , A. Verdini , L. Floreano , M. Stredansky , E. Vesselli , C. Hohner , M. Kettner , J. Libuda , C. M. Schneider , G. Zamborlini , M. Cinchetti , V. Feyer , Small 2021, 17, 2104779. 10.1002/smll.202104779 PMC11475682 34643036

[36] H. M. Sturmeit , I. Cojocariu , M. Jugovac , A. Cossaro , A. Verdini , L. Floreano , A. Sala , G. Comelli , S. Moro , M. Stredansky , M. Corva , E. Vesselli , P. Puschnig , C. M. Schneider , V. Feyer , G. Zamborlini , M. Cinchetti , J. Mater. Chem. C 2020, 8, 8876–8886.

[37] P. S. Deimel , R. M. Bababrik , B. Wang , P. J. Blowey , L. A. Rochford , P. K. Thakur , T. L. Lee , M. L. Bocquet , J. V. Barth , D. P. Woodruff , D. A. Duncan , F. Allegretti , Chem. Sci. 2016, 7, 5647–5656. 30034702 10.1039/c6sc01677d PMC6022009

[38] I. Thomas S. Rush, Pawel M. Kozlowski, Christine A. Piffat, Ranjit Kumble, Marek Z. Zgierski, Thomas G. Spiro, T. S. Rush, P. M. Kozlowski, C. A. Piffat, R. Kumble, M. Z. Zgierski, T. G. Spiro, *J. Phys. Chem. B* **2000**, *104*, 5020–5034.

[39] X. Y. Li , R. S. Czernuszewicz , J. R. Kincaid , Y. O. Su , T. G. Spiro , J. Phys. Chem. 1990, 94, 31–47.

[40] L. Scudiero , D. E. Barlow , K. W. Hipps , J. Phys. Chem. B 2000, 104, 11899–11905.

[41] N. Sheppard , C. D. La Cruz , Phys. Chem. Chem. Phys. 2010, 12, 2275–2284. 20449339 10.1039/b914016f

[42] M. Minissale , G. Fedoseev , E. Congiu , S. Ioppolo , F. Dulieu , H. Linnartz , Phys. Chem. Chem. Phys. 2014, 16, 8257–8269. 24671412 10.1039/c3cp54917h

[43] G. Di Santo , C. Castellarin-Cudia , M. Fanetti , B. Taleatu , P. Borghetti , L. Sangaletti , L. Floreano , E. Magnano , F. Bondino , A. Goldoni , J. Phys. Chem. C 2011, 115, 4155–4162. 10.1063/1.4798934 24981539

[44] K. Diller , F. Klappenberger , M. Marschall , K. Hermann , A. Nefedov , C. Wöll , J. V. Barth , J. Chem. Phys. 2012, 136, 014705. 22239798 10.1063/1.3674165

[45] L. Dalstein , A. Revel , C. Humbert , B. Busson , J. Chem. Phys. 2018, 148, 134701. 29626883 10.1063/1.5021553

[46] B. Busson , L. Dalstein , J. Chem. Phys. 2018, 149, 034701. 30037239 10.1063/1.5027154

[47] B. Busson , L. Dalstein , J. Chem. Phys. 2018, 149, 154701. 30342449 10.1063/1.5047098

[48] M. H. Matloob , M. W. Roberts , J. Chem. Soc. Faraday Trans. 1 1977, 73, 1393.

[49] M. J. Braithwaite , R. W. Joyner , M. W. Roberts , Faraday Discuss. Chem. Soc. 1975, 60, 89.

[50] R. Burch , S. T. Daniells , P. Hu , J. Chem. Phys. 2002, 117, 2902–2908.

[51] J. Gwak , S. Ahn , M.-H. Baik , Y. Lee , Chem. Sci. 2019, 10, 4767–4774. 31160953 10.1039/c9sc00717b PMC6510316

[52] D. Denysenko , D. Volkmer , Faraday Discuss. 2017, 201, 101–112. 28608896 10.1039/c7fd00034k

[53] C. Sun , L. Yang , M. A. Ortuño , A. M. Wright , T. Chen , A. R. Head , N. López , M. Dincă , Angew. Chem. Int. Ed. 2021, 60, 7845–7850; 10.1002/anie.202015359 33645907

[54] S. Metz , Inorg. Chem. 2017, 56, 3820–3833. 28291346 10.1021/acs.inorgchem.6b02551

[55] E. A. Wade , J. I. Cline , K. T. Lorenz , C. Hayden , D. W. Chandler , J. Chem. Phys. 2002, 116, 4755.

[56] M. D. Brookes , A. R. W. McKellar , T. Amano , J. Mol. Spectrosc. 1997, 185, 153–157. 9344806 10.1006/jmsp.1997.7357

[57] Y.-L. Zhao , M. D. Bartberger , K. Goto , K. Shimada , T. Kawashima , K. N. Houk , J. Am. Chem. Soc. 2005, 127, 7964–7965. 15926791 10.1021/ja042247s

[58] H. A. Duarte , E. Proynov , D. R. Salahub , J. Chem. Phys. 1998, 109, 26–35.

[59] K. A. Nguyen , M. S. Gordon , J. A. J. Montgomery , H. H. Michels , J. Phys. Chem. 1994, 98, 10072–10078.

[60] M. A. Vincent , I. H. Hillier , L. Salsi , Phys. Chem. Chem. Phys. 2000, 2, 707–714.

[61] F. Fuster , C. Dézarnaud-Dandine , H. Chevreau , A. Sevin , Phys. Chem. Chem. Phys. 2004, 6, 3228–3234.

[62] H. Tsukahara , T. Ishida , Y. Todoroki , M. Hiraoka , M. Mayumi , Free Radical Res. 2003, 37, 171–177. 12653205 10.1080/1071576021000036489

