# Supplementary material for: Disproportionation of Nitric Oxide at a Surface‐Bound Nickel Porphyrinoid
Source: Angew Chem Weinheim Bergstr Ger. 2022 Mar 21;134(20):e202201916. doi: 10.1002/ange.202201916 (PMC10947138; doi:10.1002/ange.202201916)
Supplement: Supplementary file 1 — Supporting Information [file ANGE-134-0-s001.pdf]

Angewandte Chemie
Eine Zeitschrift der Gesellschaft Deutscher Chemiker

Supporting Information

Disproportionation of Nitric Oxide at a Surface-Bound Nickel Porphyrinoid

M. Stredansky, S. Moro, M. Corva, H. Sturmeit, V. Mischke, D. Janas, I. Cojocariu, M. Jugovac, A. Cossaro, A. Verdini, L. Floreano, Z. Feng, A. Sala, G. Comelli, A. Windischbacher, P. Puschnig, C. Hohner, M. Kettner, J. Libuda, M. Cinchetti, C. M. Schneider, V. Feyer, E. Vesselli, G. Zamborlini**

Disproportionation of Nitric Oxide at a Surface-Bound Nickel Porphyrinoid.

Matus Stredansky,[1,2] Stefania Moro,[1] Manuel Corva,[1,2] Henning Sturmeit,[3] Valentin Mischke,[3] David Janas,[3] Iulia Cojocariu,[4] Matteo Jugovac,[4] Albano Cossaro,[2,5] Alberto Verdini,[2] Luca Floreano,[2] Zhijing Feng,[1,2] Alessandro Sala,[2] Giovanni Comelli,[1,2] Andreas Windischbacher,[6] Peter Puschnig,[6] Chantal Hohner,[7] Miroslav Kettner,[7] Jörg Libuda,[7] Mirko Cinchetti,[3] Claus Michael Schneider,[4,8] Vitaliy Feyer,[4,8] Erik Vesselli,[1,2,*] and Giovanni Zamborlini[3,4,*]

[1] Physics Department, University of Trieste, via A. Valerio 2, 34127 Trieste (Italy)

[2]CNR-IOM, Area Science Park, S.S. 14 km 163,5, 34149 Trieste (Italy)

[3] Department of Physics, TU Dortmund University, Dortmund (Germany)

[4]Peter Grünberg Institute (PGI-6), Forschungszentrum Jülich GmbH, Jülich (Germany)

[5]Department of Chemistry and Pharmaceutical Science, University of Trieste, via L- Giorgieri 1, 34127 Trieste (Italy)

[6]Institut für Physik, Karl-Franzens-Universität Graz, 8010 Graz (Austria)

[7]Erlangen Center for Interface Research and Catalysis, Friedrich-Alexander-Universität Erlangen-Nürnberg, Egerlandstr. 3, 91058 Erlangen (Germany)

[8]Fakultät f. Physik and Center for Nanointegration Duisburg-Essen (CENIDE), Universität Duisburg-Essen, D-47048 Duisburg (Germany)

*Corresponding authors: evesselli@units.it; giovanni.zamborlini@tu-dortmund.de;

METHODS

InfraRed-Visible Sum-Frequency Generation spectroscopy (IR-Vis SFG). A UHV system with a base pressure of 5×10^{-11} mbar hosts standard surface science preparation and characterization techniques and is directly connected to a high-pressure reactor cell for *in situ* IR-Vis SFG spectroscopy. The excitation source (Ekspla) delivers a 532 nm (2.33 eV, 30 ps, 50 Hz) visible beam and tunable IR radiation in the 1000-4500 cm^{-1} range. The SFG spectra were normalized by the non-resonant signal generated by a GaAs crystal in order to account for modulations in the IR intensity, both intrinsic and due to gas phase adsorption along the optical path. The data were then analyzed by least-squares fitting to a parametric, effective expression of the nonlinear second-order susceptibility:[1,2]

$$\frac{I_{SFG}(\omega_{IR})}{I_{vis} I_{IR}(\omega_{IR})} \propto \left| A_{NRes} + \sum_k \frac{A_k e^{i\Delta\varphi_k}}{\omega_{IR} - \omega_k + i\Gamma_k} \right|^2$$

In the manuscript's figures, we plot the normalized IR-Vis SFG signal intensity together with the best fit. For better visual clarity, we also plot (filled profiles) the intensity of each kth resonance and its interference with the nonresonant background, calculated with the parameters obtained from the fitting procedure, according to the following equation from the literature:[2]

$$\frac{I_{SFG,k}(\omega_{IR})}{I_{vis} I_{IR}(\omega_{IR})} \propto \left| A_{NRes} + \frac{A_k e^{i\Delta\varphi_k}}{\omega_{IR} - \omega_k + i\Gamma_k} \right|^2$$

InfraRed Absorption-Reflection Spectroscopy (IRAS). The IRAS measurements were performed in an UHV environment with a base pressure of 1.5×10^{-10} mbar. A detailed description of the UHV setup, which hosts standard surface characterization tools, can be found elsewhere.[3] For the acquisition of the IRAS spectra, a Fourier-transform infrared (FTIR) spectrometer (Bruker Vertex 80v) is used. The spectrometer and an external liquid-nitrogen-cooled-mercury−cadmium-telluride (LN-MCT) detector are connected to the setup via differentially pumped KBr windows. Background spectra were recorded with an acquisition time of 10 min both on the clean Cu(100) surface and on the NiTPP film, serving as a reference for the NO and NO_2 adsorption experiments (acquisition time 1 min). All spectra were recorded with a resolution of 4 cm^{-1}.

Valence Band (VB) spectroscopy. The valence band spectra and the momentum maps were measured using two different electrostatic photoemission electron microscope (PEEM) setups. One is a commercial NanoESCA (Focus GmbH) and is installed at the NanoESCA beamline of Elettra, the Italian synchrotron radiation facility in Trieste (base pressure better than 5×10^{-11} mbar).[4] The other is a commercial KREIOS set up (Specs GmbH) installed at the TU Dortmund University (base pressure better than 1×10^{-10} mbar). Both setups are connected to a sample preparation chamber equipped with standard surface characterization tools. In both PEEM setups, two-dimensional momentum maps are collected in a single-shot experiment, with a wide reciprocal

space range: k_x, $k_y \in \pm 2.1$ Å^{-1}. By means of a hemispherical electron analyzer, it is possible to select a specific kinetic energy of the photoemitted electrons, thus measuring the full energy *vs.* parallel momentum data cube. For the experiments performed with the NanoESCA microscope, the data were collected with a photon energy of 30 eV and a total energy resolution of 70 meV, using linearly p-polarized light, while keeping the sample at 90 K during the measurements. To avoid radiation damage of the molecular layer induced by the micro-focused spot provided by the beamline, the surface was raster-scanned during the acquisition (v_{raster} = 1µm/s). In the KREIOS setup, instead, experiments were carried out with a non-monochromatized He lamp (photon energy 21.2 eV), while the sample was kept at room temperature. The energy resolution provided by the instrument was 80 meV. Valence band scans were always performed on fresh areas of the surface, i.e. not previously irradiated with the UV light.

Scanning Tunneling Microscopy (STM). STM measurements were carried out in UHV with an Omicron low-temperature STM (LT-STM) cooled down to the boiling point of liquid helium (4 K). The cleaning procedure and the molecule deposition process followed the recipes developed for the XPS experiment. The NO exposure was performed at RT in the preparation chamber by gas injection through a leak valve. Tunneling current was obtained with the inverse voltage applied to the tip and grounded sample. Topographic images (constant-current mode) were processed by subtracting a background plane. Vibrational d^2I/dV^2 maps were obtained in constant-height mode by modulating the bias voltage at 4.0 mV_{rms} amplitude and 470 Hz, and acquiring the current modulation via a lock-in amplifier set at the second harmonic frequency with an integration time of 12 ms/pixel. Before any constant-height raster, the tipo was first positioned over the NiTPP center with bias +20 mV and tunneling current 20 pA. Then, the feedback circuit was set off and the bias changed.

X-Ray Photoelectron Spectroscopy (XPS). XPS experiments were performed at the ALOISA beamline of the CNR-IOM,[5] located at the Elettra synchrotron radiation facility in Trieste, Italy. Data were collected using a hemispherical electron energy analyzer in normal and off-normal emission geometries, keeping the surface at a constant, grazing angle of 4° in quasi *p*-polarization. The N and O 1*s*, and the Ni 2*p* photoemission core levels spectra were measured at photon energies of 515, 650, and 1000 eV, with a total energy resolution of 165, 250 and 500 meV, respectively. The XPS binding energies (BE) were calibrated using the Fermi level of the Cu(100) substrate. The adsorbed NO_2/NiTPP/Cu(100) molecular layers were found to be sensitive to the beam exposure during the photoelectron measurement, thus the sample position was changed after each XPS scan (photon beam transverse width of ~100 µm). The binding energy, as well as the full width half maximum (FWHM) of the Ni 2*p* core level spectrum, were determined using a Doniach-Šunjić lineshape,[6] convoluted with a Gaussian envelope that accounts for energy resolution, temperature, and heterogeneity. A Voigt lineshape was adopted instead for the 1*s* levels of the adsorbed species.

Sample preparation. In all experiments, the clean Cu(100) surface was prepared by cycles of Ar^+ ion sputtering at 2.0 keV, followed by annealing at 800 K. Depending on the system, the bare surface was checked by XPS, low-energy electron diffraction (LEED) or IRAS. A few mg of

NiTPP (Porphyrin Systems) were loaded into a quartz crucible of a home-made Knudsen cell type evaporator connected to a separate preparation chamber. Before the experiments, the molecules were carefully outgassed at 480 K for several hours while the UHV system base pressure was monitored. The molecules were thermally evaporated at ~520 K onto the copper substrate kept at room temperature. For the XPS and IR-Vis SFG measurements, the molecular coverage has been calibrated with a quartz microbalance. The ordering of the surface and of the molecular layer was checked using LEED in the case of IR-Vis SFG measurements.

NO exposure. *KREIOS set-up*: a 99.5% NO bottle was attached to a gas line connected to a high-precision leak valve. The gas line was baked prior to the experiment at 360 K for 20 h. Before the first use for the uptake after the bake out, the gas line was flushed several times with NO. Moreover, part of the gas line was going through a cryo-trap kept at 200 K, in order to further purify the content of the NO bottle. The gas line was emptied after each uptake experiment via a dedicated pumping station equipped with a turbopump (vacuum better than 1×10^{-6} mbar). The NO dose was performed in the KREIOS preparation chamber, and the pressure was measured with a cold cathode gauge. During the exposure all the *in vacuo* devices with a filament were switched off, to avoid cracking/reaction of the NO molecule followed by the recombination in undesired products. *SFG*: NO was dosed from a dedicated gas line with aluminum pipes that was previously pumped and repeatedly flushed with NO. The uptakes were performed in the measurement cell. Pressure was monitored by means of a pyrex sniffer connected to the (separated) main chamber, into which the cell atmosphere was injected, and where an ion gauge and a mass spectrometer were operated, thus avoiding any influence on the gas by pressure measurement and gas composition monitoring. The pressure ratio between the reaction cell and the main chamber was previously calibrated. *IRAS*: NO and NO_2 were both dosed via an effusive beam and a PC-controlled inlet valve. For NO, the pressure was set to 1×10^{-6} mbar, thus only the valve opening time was tuned to achieve the desired exposure. A cold trap was used to further purify NO. *NanoESCA, STM and ALOISA*: the NO bottle (Linde, 99.5%) was attached to a gas line connected to a high-precision leak valve. The gas line was baked prior to the experiment at 360 K for 20 h. After the bake out, it was flushed several times with NO before the uptake.

Density Functional Theory (DFT). All calculations were performed within the framework of DFT. We utilized the Vienna Ab Initio Simulation Package (VASP) version 5.4.4 for periodic systems as well as for isolated molecules.[7,8] Exchange-correlation effects were described by the functional of Perdew-Burke-Ernzerhof,[9] and van der Waals contributions treated with the D3 dispersion correction.[10] The projector augmented wave method was used together with an energy cutoff of 400 eV.[11] All calculations were spin-polarized and the ionic positions optimized until forces were below 0.01 eV/Å. The surfaces (Cu and Cu-O) were simulated within the repeated slab approach using four substrate layers, two of which were held fix during optimization, and a 25 Å vacuum layer between the slabs. To avoid spurious electrical fields, a dipole layer was inserted in the vacuum region.[12] The periodic surface calculations were obtained on a Monkhorst-Pack 4×4×1 grid of k-points.[13] The isolated molecules were calculated in a sufficiently big unit cell ensuring a minimum of 15 Å of vacuum to each side only using the Γ-point of the BZ. In

addition, isolated molecules in the gas phase were also calculated with the quantum chemistry package ORCA version 4.2.1.[14] Molecular geometries were optimized on three different levels: PBE,[9] in combination with the def2-TZVP basis set,[15] the hybrid functional B3LYP,[16,17] in combination with the def2-TZVP basis set,[15] and second order Møller–Plesset perturbation theory (MP2) in combination with the cc-pVTZ basis set.[18–20] All systems were calculated as open-shell systems with the respective multiplicity. Dispersion was accounted for by Grimme's D3 correction.[10]

Supplementary Figures and Tables

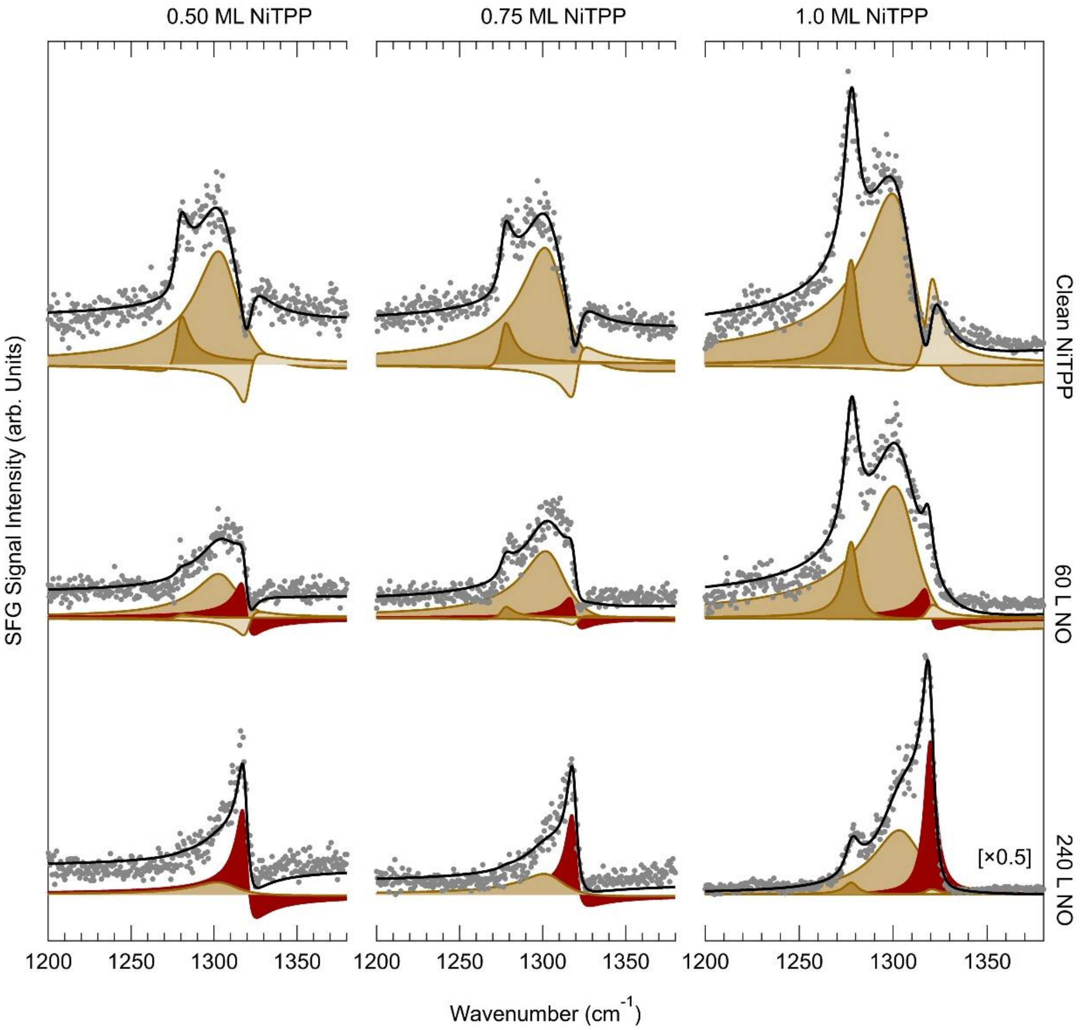

Figure S1. IR-Vis SFG spectra of 0.50, 0.75, and 1.0 ML NiTPP/Cu(100) of the pristine layers and after exposure at room temperature to 60 and 240 L of NO; data are shown (grey markers) together with the best fit (black lines) and deconvolution (filled profiles) obtained according to the lineshape described in the text.

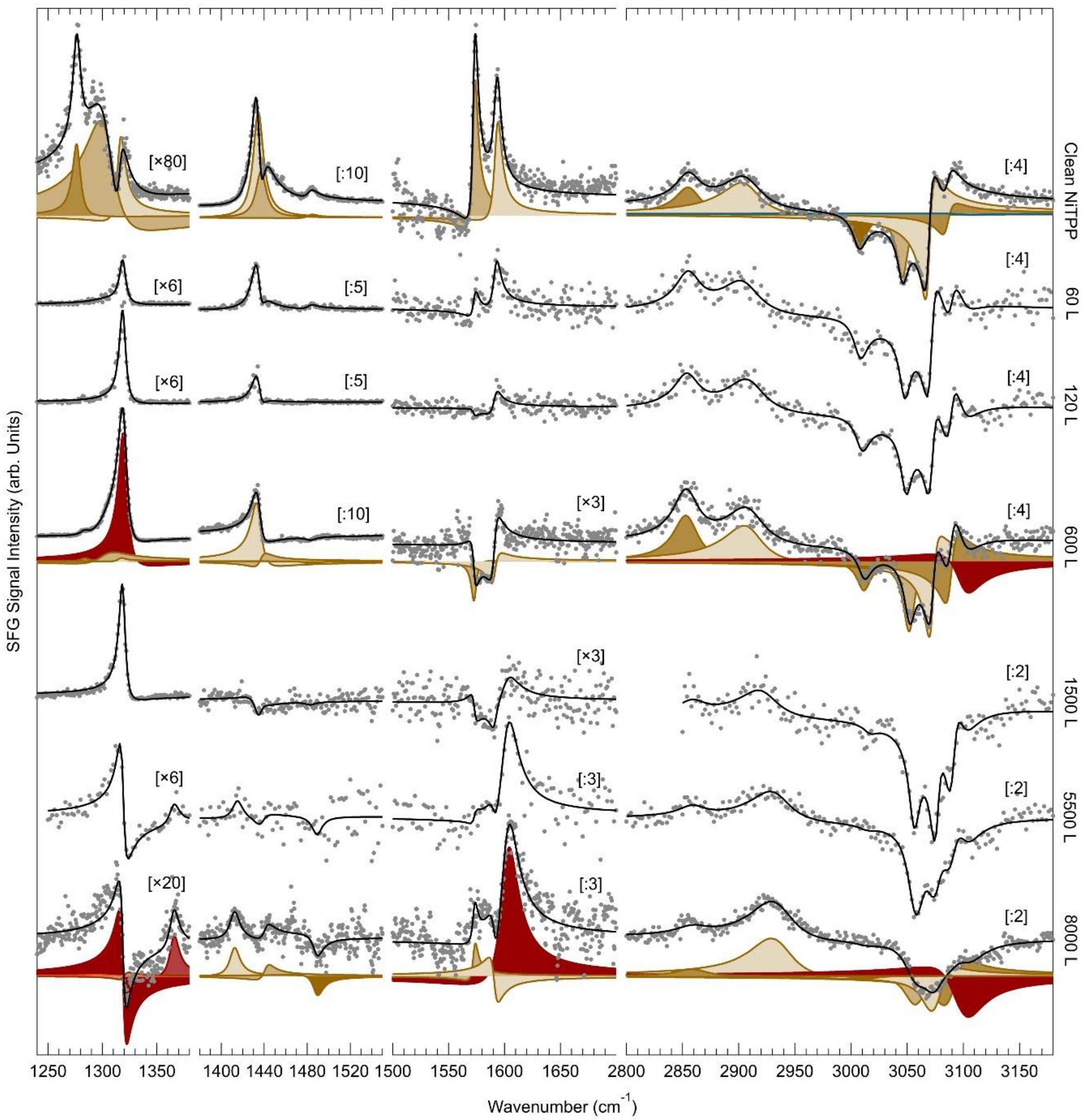

Figure S2. IR-Vis SFG spectra of 1.0 ML NiTPP/Cu(100) as a function of the NO dose at room temperature; data are shown (grey markers) together with the best fit (black lines) and deconvolution (filled profiles) obtained according to the lineshape described in the text.

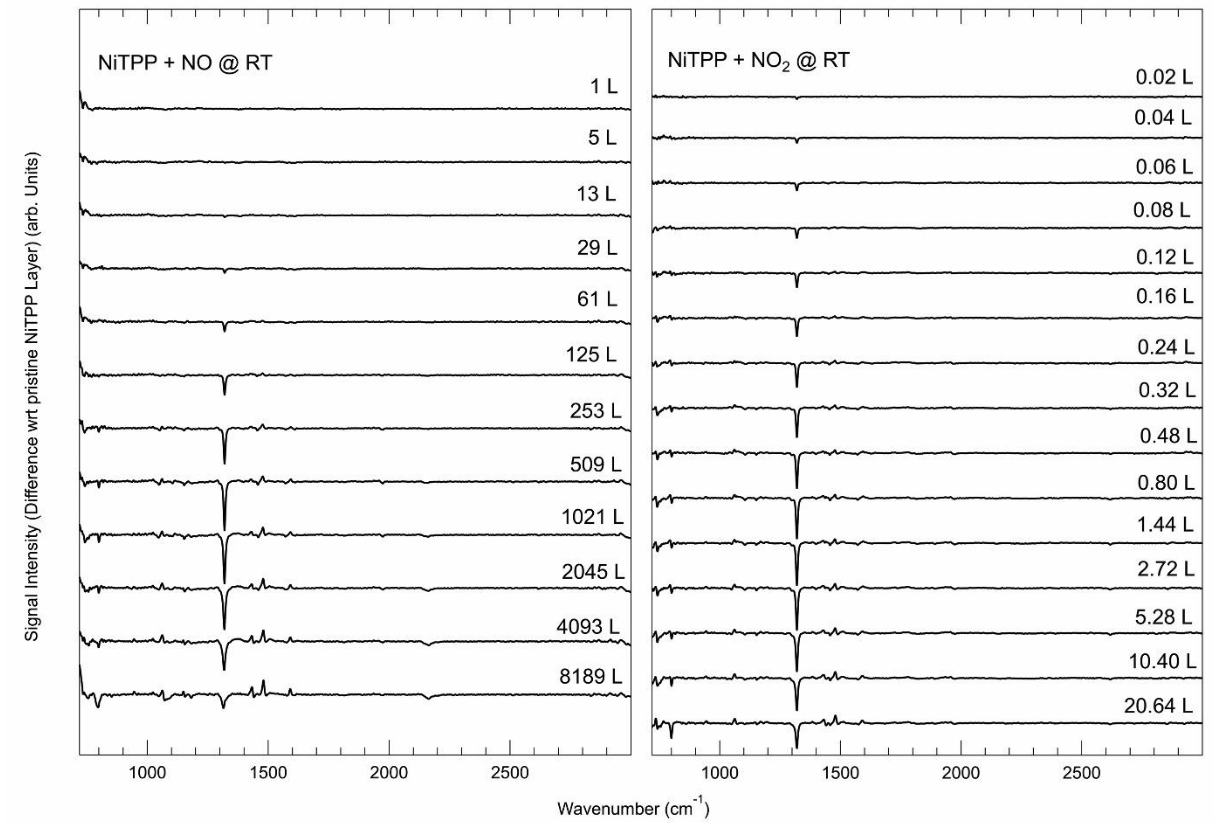

Figure S3. Difference IRAS spectra (with respect to the pristine NiTPP/Cu(100) monolayer) as a function of the NO (left) and NO_2 (right) exposure at room temperature.

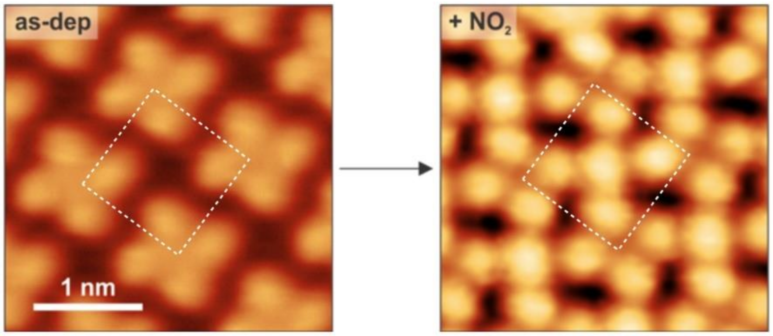

Figure S4. STM images of NiTPP/Cu(100) network before (left) and after (right) exposure to NO_2.[21] A single NiTPP molecule is framed with a dashed white square to guide the eye. All images are acquired at 77 K. Tunnelling parameters: (left) V_b = -1.0 V, I = 500 pA, (right) V_b = +1.0 V, I = 200 pA.[21]

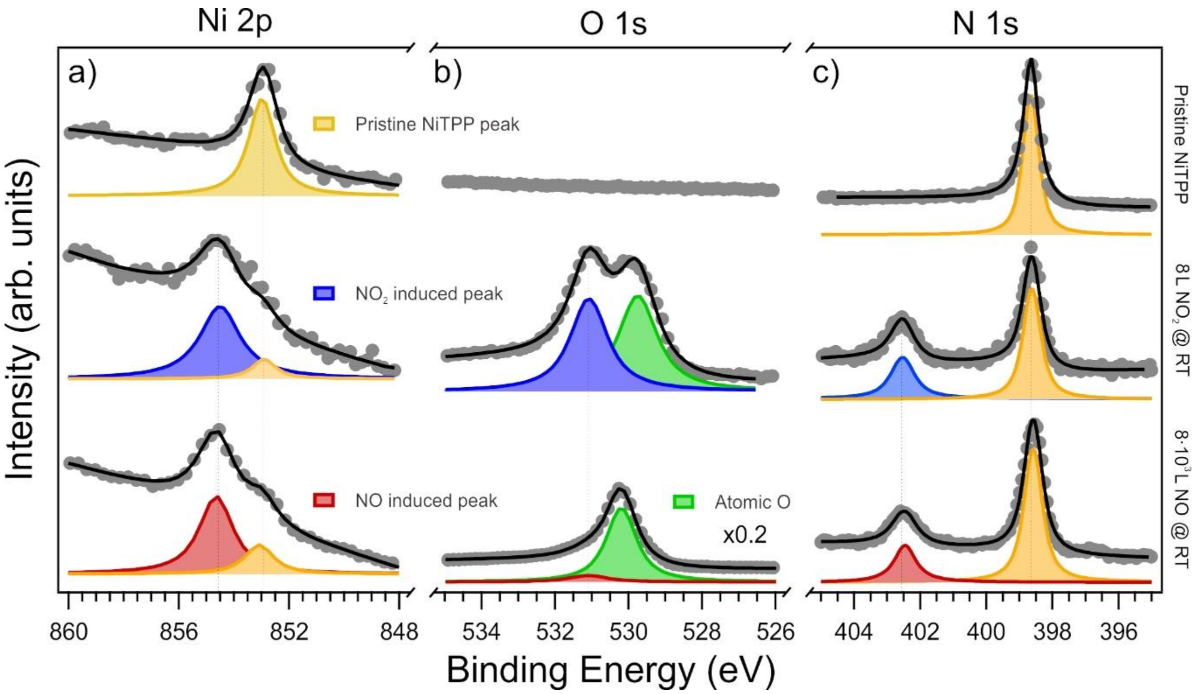

Figure S5. Ni 2$p_{3/2}$ (left) and O (center) and N (right) 1*s* XPS core level spectra of the pristine NiTPP/Cu(100) layer (top) and after exposure to 8 L of NO_2 (middle) and to 8×10^3 L of NO (bottom) at room temperature, plotted together with the corresponding fit. All spectra were collected in a normal emission geometry, with photon energies of 1050 eV (Ni 2*p*), 650 eV (O 1*s*) and 515 eV (N 1*s*).

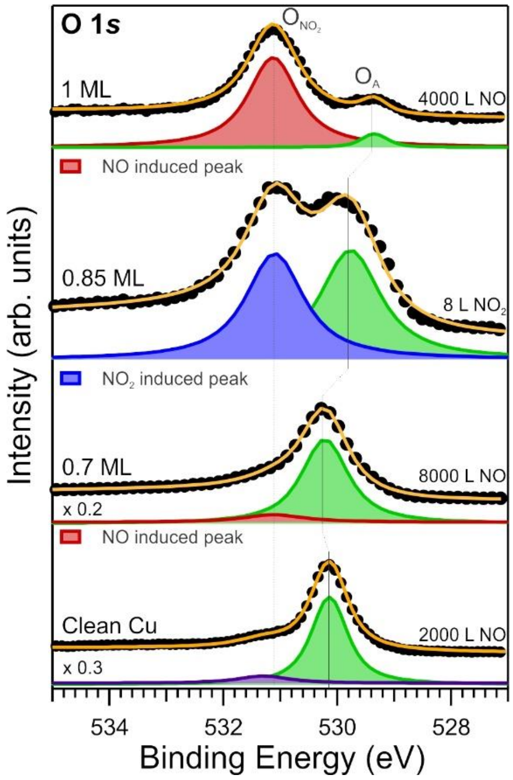

Figure S6. O 1*s* core level photoemission spectra (experimental data, overall fits and single fit components) of NiTPP/Cu(100) layers upon exposure to 2000 and 4000 L of NO, compared with the exposure to NO_2 and of the bare Cu(100) termination to NO (bottom). The spectra were taken with 650 eV photon energy.

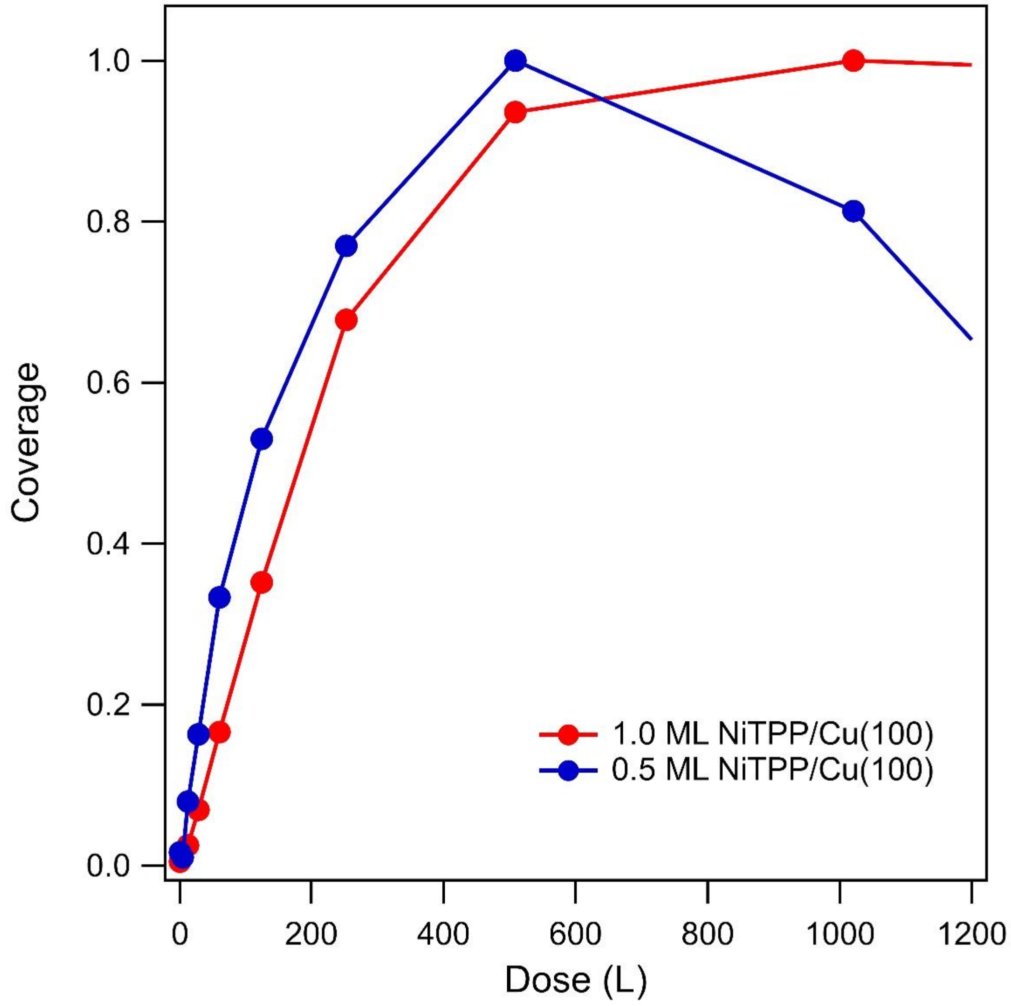

Figure S7. Normalized IRAS peak intensity of ν_s(ONO) as a function of the NO exposure for different initial NiTPP film coverages. To further support the fact that NO disproportionation occurs at the Ni centers within the porphyrin macrocycle rather than on the residual bare copper areas, we performed the NO uptake on 0.5 and 1.0 ML NiTPP, respectively, monitoring the intensity of the ν_s(ONO) mode as a function of the NO exposure, by means of *in-situ* IRAS. The signal was normalized to saturation, corresponding to the situation where all available Ni centers are coordinated with an NO_2 molecule. Qualitatively, the two uptake curves do not differ significantly, while they show a different decay behavior at high NO exposure, showing that bare Cu contributes with NO dissociation, formation of atomic O, progressive oxidation of the substrate and, consequently, decoupling of the NiTPP layer, making the molecular film inert. As a consequence, the ligation of NO_2 to the Ni center is hindered, leading to a weaken in the intensity of the ν_s(ONO) mode, in agreement with the results obtained by means of the other techniques as described in the main text.

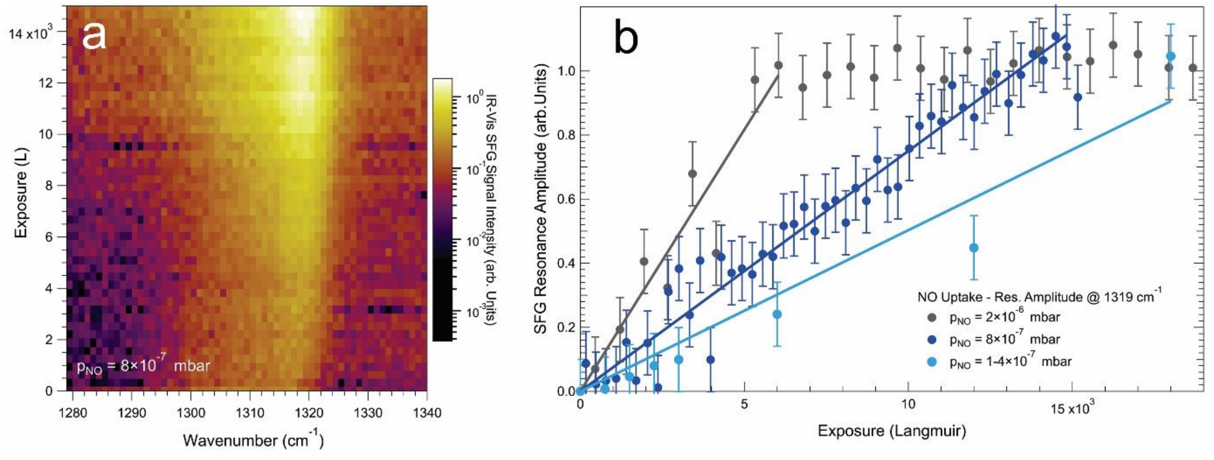

Figure S8. Time-resolved IR-Vis SFG data of NO uptakes on 1 ML NiTPP/Cu(100) performed at room temperature with different NO background pressures: (a) IR-Vis SFG intensity map (color scale) as a function of the NO exposure (left axis) and of the IR wavelength (bottom axis); (b) amplitude evolution of the NO-induced IR-Vis SFG resonance at 1319 cm^{-1} (asymmetric NO_2 stretching) as a function of the NO exposure for different background pressures as obtained by least-square fitting of the spectroscopic data collected *in situ* like the series reported in (a).

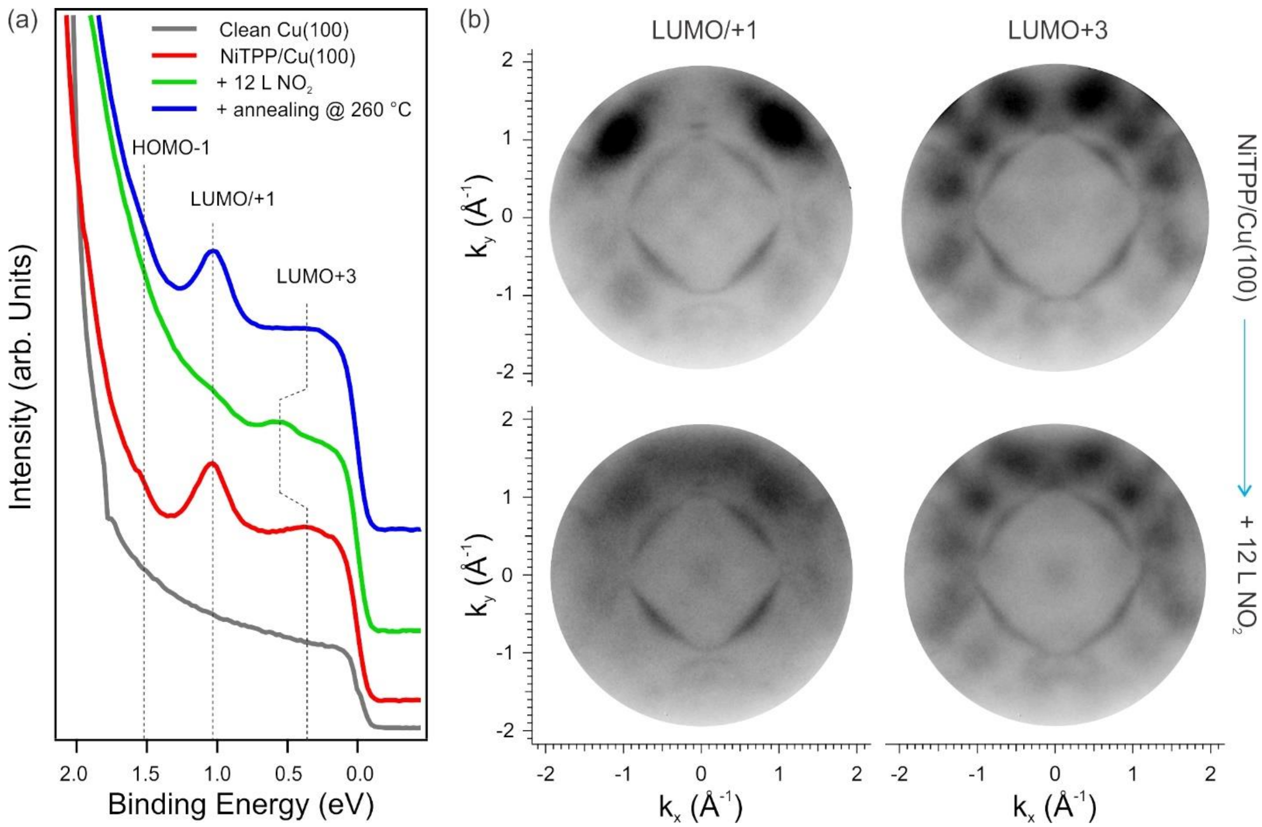

Figure S9 (a) Quenching of the LUMO/+1 VB feature upon exposure of the NiTPP/Cu(100) system to NO_2; the effect is reversible upon annealing (desorption of NO_2); (b) clear fingerprint in momentum space associated with the LUMO+3 and the degenerate LUMO and LUMO+1 of the adsorbed NiTPP of the molecular features evidenced in (a).

In order to extract the NO_2 formation rate as a function of the NO pressure during the uptake, we tracked the evolution of the valence band features related to the NiTPP molecular orbitals upon a stepwise NO exposure. In a control experiment, we exposed the NiTPP/Cu(100) to 12 L of NO_2 and we followed the changes in the valence band. In a later stage, we annealed the system up to 530 K in order to remove NO_2 and restore the molecular layer. The resulting angle-integrated valence band spectra, as well as the momentum maps taken at a specific binding energy, are reported in Figure S8. While the valence band (VB) of the clean copper (Figure S9a, grey curve) shows a rather featureless plateau, the NiTPP/Cu(100) spectrum (red curve) has two prominent features at binding energies of 0.3 and 1.0 eV. These features have a clear fingerprint in momentum space (Figure S9b, top row) and we associated to the LUMO+3 and the degenerate LUMO and LUMO+1 (LUMO/+1 in the following) of the adsorbed NiTPP, respectively.[22] Upon NO_2 exposure, we witness a quenching of the LUMO/+1 feature (see Figure S9a, green curve), and, at the same time, the LUMO+3 moves shift to a higher binding energy (0.5 eV). The LUMO/+1 quenching can also be appreciated in the intensity decrease of the corresponding momentum map, which is shown in Figure S8b, bottom left. On the other hand, the intensity of LUMO+3 is almost

unaffected by the interaction of the NO_2 with the NiTPP, suggesting that the macrocycle is still strongly hybridized with the underlying substrate.[21] In our previous works we proved that the interface is extremely robust against thermal treatments,[23] and heating up to 390 K is sufficient to trigger the NO_2 desorption. For these reasons we annealed the NO_2-NiTPP/Cu(100) up to 530 K in order to remove the nitrogen dioxide. The corresponding VB spectrum is reported in Figure S8a, blue curve. The annealing results in a complete restoring of the LUMO/+1 intensity, as well as back shift of the LUMO+3 binding energy to its original value. These evidences suggest that the intensity of LUMO/+1 resonance can be directly related to the NO_2 adsorption process. In the following, we will show that this quantity can be used also to quantify the amount of NiTPP molecules coordinated to NO_2 and thus, when the molecular interface is exposed to NO, it can be exploited to obtain the NO_2 formation rate.

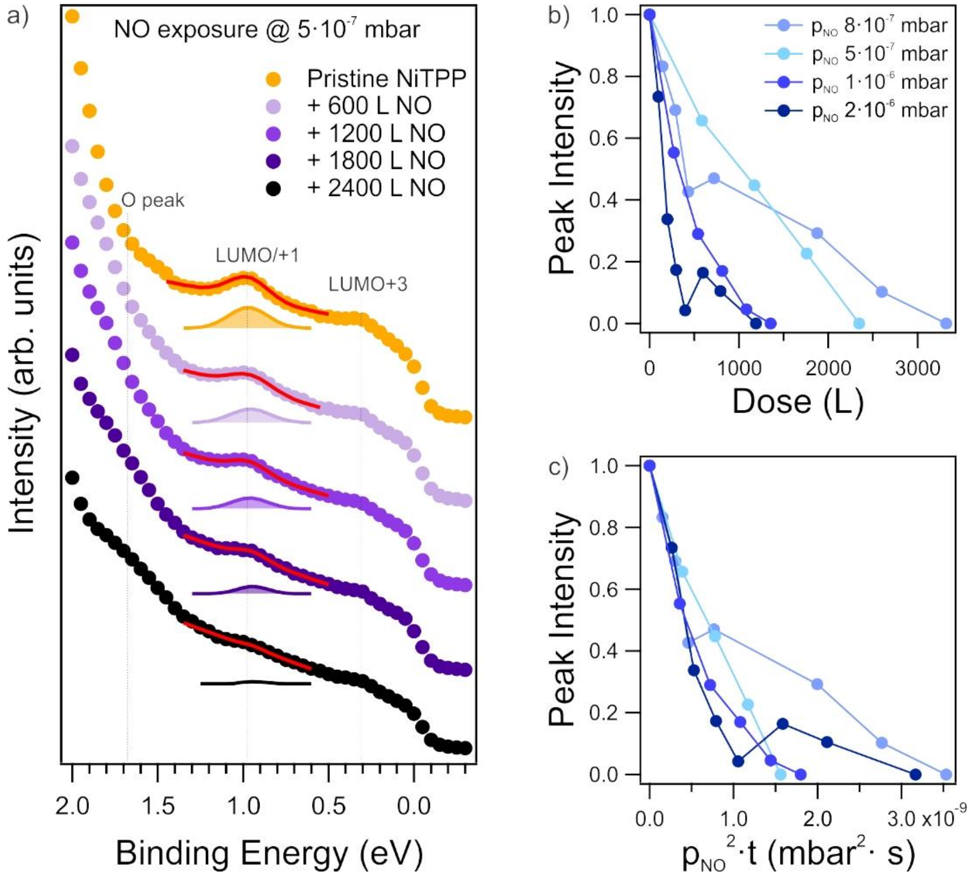

Figure S10. (a) Valence band measurements of the NiTPP/Cu(100) interface exposed to increasing doses of NO. For each spectrum the total dose is indicated. The NO pressure was kept to 5×10^{-7} mbar during the exposures. An unpolarized He(I) source, with a photon energy of 21.2 eV was used. (b) The peak intensity of the LUMO/+1 feature is plotted as a function of the NO dose, for different NO uptake pressures. (c) The same peak intensity displayed in (b) is plotted as a function of the square of the NO pressure multiplied by the exposure time.

Although the microscope is capable of acquiring momentum maps with high k resolution, the limitations due to the unpolarized He(I) source and the available photon energy (21.2 eV) did not allow us to see any clear fingerprint of the molecular orbital in momentum space. For this reason, in Figure S10 only the angle-integrated spectra, together with the corresponding analysis, are reported. The pristine NiTPP film shows a VB spectrum (yellow curve) fully compatible with the one reported in Figure S8a. The two molecular resonances, i.e. the LUMO/+1 and LUMO+3, can be identified at the same BEs. Upon NO exposure, the feature related to the LUMO/+1 decreases in intensity, and it almost vanishes after dosing 2400 L. At the same time, a new component rises at around 1.7 eV. We tentatively associate it to the adsorbed/intercalated oxygen resulting from the dissociation of NO on bare copper surface. The decrease of the LUMO/+1 peak was quantified by fitting the corresponding region of the spectrum with a Gaussian function and a linear background. The experiment was repeated for different uptake pressures of NO, namely $5 \cdot 10^{-7}$,

$8 \cdot 10^{-7}$, $1 \cdot 10^{-6}$ and $2 \cdot 10^{-6}$ mbar. The results were then plotted either as a function of the dose (Figure S10b) or as a function of the square of the pressure multiplied by the corresponding exposure time (Figure S10c). In the former case each uptake curve is well separated from the others, reaching saturation at a different dose. In the latter, instead, the curves almost overlap at the beginning of the uptake, suggesting that the formation of NO_2 by dosing NO is quadratic in the NO pressure. By fitting with a line the first part of the uptake, we can extract the NO_2 formation rates (in ML/s). The latter are reported in the main text in Figure 2, together with the corresponding data obtained by means of IR-Vis SFG.

		UHV	60L	120L	360L	600L	1500L	2500L	5500L	7500L	8500L
	A(NR)	0.09	0.04	0.07	0.17	0.19	0.21	0.42	0.53	0.78	0.8
**P1**	A	0.40	0	0	0	0	0	0	0	0	0
	Δφ	259	259	259	259	259	259	259	259	259	259
	Ω	1277	1277	1277	1277	1277	1277	1277	1277	1277	1277
	Γ	4.5	4.5	4.5	4.5	4.5	4.5	4.5	4.5	4.5	4.5
**P2**	A	2.9	1.2	0.08	0	0	0	0	0	0	0
	Δφ	330	330	330	330	330	330	330	330	330	330
	Ω	1305	1305	1305	1305	1305	1305	1305	1305	1305	1305
	Γ	16	16	16	16	16	16	16	16	16	16
**P3**	A	0.31	0.11	0	0	0	0	0	0	0	0
	Δφ	180	180	180	180	180	180	180	180	180	180
	Ω	1320	1320	1320	1320	1320	1320	1320	1320	1320	1320
	Γ	4.5	4.5	4.5	4.5	4.5	4.5	4.5	4.5	4.5	4.5
**NO1**	A	0	1.6	2.5	4.3	4.2	3.8	3.6	2.3	1.6	1.7
	Δφ	7	7	7	7	7	10	16	19	19	19
	Ω	1319	1319	1319	1319	1319	1319	1319	1319	1319	1319
	Γ	3.5	3.5	3.5	3.5	3.5	3.5	3.5	3.5	3.5	3.5
**NO2**	A	0	0	0	0	0	0	0	0.58	0.85	0.71
	Δφ	250	250	250	250	250	250	250	250	250	250
	Ω	1365	1365	1365	1365	1365	1365	1365	1365	1365	1365
	Γ	5.4	5.4	5.4	5.4	5.4	5.4	5.4	5.4	5.4	5.4
	A(NR)	0.13	0.07	0.08	0.18	0.19	0.21	0.40	0.63	0.93	0.90
**P4**	A	12	6.1	4.5	0.92	0.85	0.20	0.48	0.43	0.76	0.80
	Δφ	246	285	310	356	356	255	260	267	267	260
	Ω	1434	1434	1434	1434	1434	1415	1415	1415	1415	1412
	Γ	5.7	5.7	5.7	5.7	5.7	5.7	5.7	5.7	5.7	5.7
**P5**	A	9.5	4.3	3.3	0.34	0.52	0.30	0.39	0.21	0.46	0.42
	Δφ	97	130	142	145	145	99	136	136	190	209
	Ω	1437	1437	1437	1437	1437	1437	1437	1437	1438	1441
	Γ	5.7	5.7	5.7	5.7	5.7	5.7	5.7	5.7	5.7	5.7
**P6**	A	0.52	0.45	0.01	0.18	0.13	0.19	0.25	0.28	0.41	0.57
	Δφ	214	230	81	81	81	110	127	86	40	64
	ω	1483	1483	1483	1483	1483	1488	1488	1488	1488	1488
	Γ	5.7	5.7	5.7	5.7	5.7	5.7	5.7	5.7	5.7	5.7

Table S1. Deconvolution parameters of the IR-Vis SFG resonances observed for the NO uptake at room temperature on 1 ML NiTPP/Cu(100) in the 1240 – 1500 cm^{-1} range. Resonances are labelled with P (pristine porphyrin) and NO (NO-induced).

		UHV	60L	120L	360L	600L	1500L	2500L	5500L	7500L	8500L
	A(NR)	0.25	0.13	0.12	0.23	0.25	0.30	0.48	0.83	1.14	1.10
**P7**	A	1.5	0.76	0.51	1.2	1.3	1.6	2.2	0.17	0.43	0.42
	Δφ	105	97	80	86	89	85	72	174	226	228
	ω	1573	1573	1573	1573	1573	1573	1573	1573	1573	1573
	Γ	2.8	2.8	2.8	2.8	2.8	2.8	2.8	2.8	2.8	2.8
**P8**	A	0.73	0.48	0.31	0.33	0.27	0.24	0.55	0.53	0.71	0.70
	Δφ	237	207	181	115	101	96	109	20	0.5	15
	ω	1593	1592	1591	1591	1591	1591	1591	1591	1591	1591
	Γ	4.1	4.1	4.1	4.1	4.1	4.1	4.1	4.1	4.1	4.1
**NO3**	A	0	0	0	0	0	0.56	2.3	4.3	4.8	4.6
	Δφ	215	215	215	215	215	215	215	215	215	215
	ω	1602	1602	1602	1602	1602	1602	1602	1602	1602	1602
	Γ	9.5	9.5	9.5	9.5	9.5	9.5	9.5	9.5	9.5	9.5

Table S2. Deconvolution parameters of the IR-Vis SFG resonances observed for the NO uptake at room temperature on 1 ML NiTPP/Cu(100) in the 1500 – 1700 cm^{-1} range. Resonances are labelled with P (pristine porphyrin) and NO (NO-induced).

		UHV	60L	120L	360L	600L	1500L	2500L	5500L	7500L	8500L
	A(NR)	0.96	0.98	0.97	0.96	0.94	0.97	0.98	0.98	0.98	0.99
	G	0 0	0	0	0	0	0	0	0	10	12
**P9**	A	1.7	2.4	2.1	2.0	1.4	-	-	0.50	-	0.31
	Δφ	280	280	280	280	280	280	280	280	280	280
	ω	2856	2856	2856	2856	2856	2856	2856	2856	2856	2856
	Γ	13.5	13.5	13.5	13.5	13.5	13.5	13.5	13.5	13.5	13.5
**P10**	A	3.1	3.8	3.5	3.8	4.2	1.5	1.2	1.8	1.6	2.5
	Δφ	295	295	295	295	295	295	295	295	295	295
	ω	2907	2907	2907	2920	2920	2920	2920	2933	2933	2933
	Γ	21	21	21	21	21	21	21	21	21	21
**P11**	A	1.5	1.6	1.6	0.6	0.7	0.2	0.3	0	0	0
	Δφ	87	87	87	87	87	87	87	87	87	87
	ω	3007	3007	3009	3009	3009	3009	3015	3015	3015	3015
	Γ	7.8	7.8	7.8	7.8	7.8	7.8	7.8	7.8	7.8	7.8
**P12**	A	2.6	3.5	3.6	2.2	1.8	1.8	1.2	1.6	0	0
	Δφ	97	97	97	97	97	97	97	97	97	97
	ω	3046	3048	3049	3053	3055	3055	3058	3058	3058	3058
	Γ	6	6	6	6	6	6	6	6	6	6
**P13**	A	3.0	3.1	3.8	2.5	2.2	2.0	1.5	1.4	3.1	1.9
	Δφ	131	131	131	131	131	131	131	131	131	131
	ω	3069	3071	3072	3074	3074	3074	3075	3075	3075	3075
	Γ	4.6	4.6	4.6	4.6	4.6	4.6	4.6	4.6	4.6	4.6
**P14**	A	0.85	0.5	1.8	3.0	2.7	2.2	2.0	1.2	1.4	1.3
	Δφ	155	155	64	64	64	64	64	64	64	64
	ω	3086	3089	3081	3081	3081	3081	3081	3081	3096	3097
	Γ	6	6	6	6	6	6	6	6	6	6
**NO4**	A	0.08	1.7	3.1	4.7	4.6	2.9	3.1	2.2	2.3	2.0
	Δφ	45	45	45	45	45	45	45	45	45	45
	ω	3097	3100	3098	3096	3097	3096	3097	3097	3097	3097
	Γ	13	13	13	13	13	13	13	13	13	13

Table S3. Deconvolution parameters of the IR-Vis SFG resonances observed for the NO uptake at room temperature on 1 ML NiTPP/Cu(100) in the 2800 – 3100 cm^{-1} range. Resonances are labelled with P (pristine porphyrin) and NO (NO-induced).

		UHV	60L	120L	240L	600L
	A(NR)	0.39	0.23	0.20	0.24	0.37
**P1**	A	0.60	0	0	0	0
	Δφ	223	223	223	223	223
	ω	1279	1279	1279	1279	1279
	Γ	4.5	4.5	4.5	4.5	4.5
**P2**	A	4.0	2.5	1.5	0.84	0.58
	Δφ	298	298	298	298	298
	ω	1305	1305	1305	1305	1305
	Γ	15.7	15.7	15.7	15.7	15.7
**P3**	A	0.69	0.58	0	0	0
	Δφ	137	137	137	137	137
	ω	1320	1320	1320	1320	1320
	Γ	4.5	4.5	4.5	4.5	4.5
**NO1**	A	0	0.80	0.87	1.4	2.7
	Δφ	7	7	7	7	7
	ω	1319	1319	1319	1319	1319
	Γ	3.5	3.5	3.5	3.5	3.5

Table S4. Deconvolution parameters of the IR-Vis SFG resonances observed for the NO uptake at room temperature on 0.50 ML NiTPP/Cu(100) in the 1200 – 1320 cm^{-1} range. Resonances are labelled with P (pristine porphyrin) and NO (NO-induced).

		UHV	15L	30L	45L	60L	120L	240L	600L
	A(NR)	0.32	0.18	0.13	0.14	0.11	0.14	0.16	0.26
**P1**	A	0.72	0.32	0.21	0.15	0.11	0	0	0
	Δφ	230	230	230	230	230	230	230	230
	ω	1277	1277	1277	1277	1277	1277	1277	1277
	Γ	4.5	4.5	4.5	4.5	4.5	4.5	4.5	4.5
**P2**	A	5.0	3.9	3.0	2.5	2.3	2.7	1.8	0.6
	Δφ	311	311	311	311	311	311 311	311	1305
	ω	1305	1305	1305	1305	1305	1305	1305	
	Γ	15.7	15.7	15.7	15.7	15.7	15.7	15.7	15.7
**P3**	A	1.1	0.77	0.48	0.39	0.31	0	0	0
	Δφ	141	141	141	141	141	141	141	141
	ω	1320	1320	1320	1320	1320	1320	1320	1320
	Γ	4.5	4.5	4.5	4.5	4.5	4.5	4.5	4.5
**NO1**	A	0	0	0.15	0.25	0.35	0.86	1.4	3.1
	Δφ	7	7	7	7	7	7	7	7
	ω	1319	1319	1319	1319	1319	1319	1319	1319
	Γ	3.5	3.5	3.5	3.5	3.5	3.5	3.5	3.5

Table S5. Deconvolution parameters of the IR-Vis SFG resonances observed for the NO uptake at room temperature on 0.75 ML NiTPP/Cu(100) in the 1200 – 1320 cm^{-1} range. Resonances are labelled with P (pristine porphyrin) and NO (NO-induced).

		N-O	N-N	N-Ni
**$(NO)_2$**	PBE	1.16	2.00	-
	B3LYP	1.15	1.98	-
	MP2	1.17	2.18	-
**$(NO)_2$-NiTPP**	PBE	1.17	2.00	2.41
	B3LYP	1.15	1.97	3.15
	MP2	1.17	2.25	2.61

Table S6. Bond lengths (Å) of $(NO)_2$ and $(NO)_2$-NiTPP in the gas phase calculated with ORCA applying different approaches (PBE, B3LYP, MP2). We want to emphasize that the accurate theoretical description of dimeric NO species is a challenging task. Standard single determinant methods such as PBE used in periodic calculations are not sufficient to describe the bonding situation. Instead, the experimentally observed geometries and dissociation energies are reproduced by post Hartree-Fock methods, where correlation is accounted for (in first approximation by perturbation theory, MP2).[24–28] Indeed, gas phase calculations applying MP2 improved the distance between the two NO moieties (2.18Å) toward the experimental value (2.26Å). However, regardless of the method the dimer does not interact with an uncharged Ni d^8 complex, while providing one additional charge to the system stabilizes the hyponitrite species. These results, therefore, encourage us that, even if energetic arguments within the pathway are difficult to base on periodic PBE calculations, the qualitative conclusions are reasonable.

As a measure for the coordination of NO_x to NiTPP, we have calculated the adsorption energies, E_{ad}, of the gases to the Ni center as $E_{ad} = E_{full} - E_{NOx} - E_{NiTPP}$, where all three subsystems have been fully relaxed (see Figure 3 and Tables S6-8). While there is no apparent trend for NiTPP complexes on the passivated Cu-surface, we see an increasingly favorable adsorption for the gases to NiTPP on the clean surface. Note that the negative adsorption energies would predict a coordinated NO_2-NiTPP complex already on the passivated surface, while this is not observed experimentally at room temperature due to the low binding energy. We have calculated the adsorption energies for the NO_2-complex on both surfaces non-self consistently with an advanced hybrid functional (Heyd-Scuseria-Ernzerhof, HSE). Especially in the case of the passivated surface, the binding gets significantly weaker (values in parentheses in Table S7). Up to date however, the optimization of large periodic systems applying hybrid functionals is prohibitively computationally expensive. As a counter test calculation, we have therefore calculated the coordination energies for the single complexes in the gas phase using the quantum chemistry code ORCA. We observe the same trend going from a GGA (PBE) to a hybrid functional (B3LYP). A further improvement in our simulation would include the consideration of temperature effects via vibrational frequencies calculations.

	NO	cis-$(NO)_2$	NO_2
VASP			
**NO_x-NiTPP/O/Cu(100)**	-0.526	-0.411	-0.639 (-0.158)
**NO_x-NiTPP/Cu(100)**	-0.927	-1.219	-1.561 (-1.479)
ORCA			
**NO_x-NiTPP (PBE)**	-0.408	-0.426	-0.620
**NO_x-NiTPP (B3LYP)**	-0.066	-0.372	-0.319

Table S7: Adsorption energies (eV) of NO_x species calculated with different level of theory. For the clean and passivated Cu surface, the PBE-D3/VASP values are reported. Periodic single point HSE-values are given in parentheses. For gas phase calculations, we utilized ORCA applying PBE-D3 and B3LYP-D3.

	3 NO	cis-$(NO)_2$ + NO	NO_2 + N_2O
**NO_x-NiTPP/O/Cu(100)**	0.000	-0.623	-2.358
**NO_x-NiTPP/Cu(100)**	0.000	-0.925	-3.763

Table S8: Electronic energies (eV) relative to the reactants for the reaction 3NO → cis-$(NO)_2$ + NO → NO_2 + N_2O at the NiTPP molecule on a clean and an oxygen-passivated Cu-surface calculated with PBE-D3/VASP.

References

[1] C. S. Tian, Y. R. Shen, *Surf. Sci. Rep.* **2014**, *69*, 105–131.

[2] M. Corva, E. Vesselli, *J. Phys. Chem. C* **2016**, *120*, 22298–22303.

[3] P. Ferstl, S. Mehl, M. A. Arman, M. Schuler, A. Toghan, B. Laszlo, Y. Lykhach, O. Brummel, E. Lundgren, J. Knudsen, L. Hammer, M. A. Schneider, J. Libuda, *J. Phys. Chem. C* **2015**, *119*, 16688–16699.

[4] C. M. Schneider, C. Wiemann, M. Patt, V. Feyer, L. Plucinski, I. P. Krug, M. Escher, N. Weber, M. Merkel, O. Renault, N. Barrett, *J. Electron Spectros. Relat. Phenomena* **2012**, *185*, 330–339.

[5] L. Floreano, A. Cossaro, R. Gotter, A. Verdini, G. Bavdek, F. Evangelista, A. Ruocco, A. Morgante, D. Cvetko, *J. Phys. Chem. C* **2008**, *112*, 10794–10802.

[6] S. Doniach, M. Sunjic, *J. Phys. C Solid State Phys.* **1970**, *3*, 285–291.

[7] G. Kresse, J. Hafner, *Phys. Rev. B* **1993**, *47*, 558–561.

[8] G. Kresse, D. Joubert, *Phys. Rev. B* **1999**, *59*, 1758–1775.

[9] J. P. Perdew, K. Burke, M. Ernzerhof, *Phys. Rev. Lett.* **1996**, *77*, 3865–3868.

[10] S. Grimme, J. Antony, S. Ehrlich, H. Krieg, *J. Chem. Phys.* **2010**, *132*, 154104.

[11] P. E. Blöchl, *Phys. Rev. B* **1994**, *50*, 17953–17979.

[12] J. Neugebauer, M. Scheffler, *Phys. Rev. B* **1992**, *46*, 16067–16080.

[13] H. J. Monkhorst, J. D. Pack, *Phys. Rev. B* **1976**, *13*, 5188–5192.

[14] F. Neese, *WIREs Comput. Mol. Sci.* **2012**, *2*, 73–78.

[15] F. Weigend, R. Ahlrichs, *Phys. Chem. Chem. Phys.* **2005**, *7*, 3297.

[16] A. D. Becke, *J. Chem. Phys.* **1993**, *98*, 5648–5652.

[17] P. J. Stephens, F. J. Devlin, C. F. Chabalowski, M. J. Frisch, *J. Phys. Chem.* **1994**, *98*, 11623–11627.

[18] T. H. Dunning, *J. Chem. Phys.* **1989**, *90*, 1007–1023.

[19] N. B. Balabanov, K. A. Peterson, *J. Chem. Phys.* **2005**, *123*, 064107.

[20] N. B. Balabanov, K. A. Peterson, *J. Chem. Phys.* **2006**, *125*, 074110.

[21] H. M. Sturmeit, I. Cojocariu, A. Windischbacher, P. Puschnig, C. Piamonteze, M. Jugovac, A. Sala, C. Africh, G. Comelli, A. Cossaro, A. Verdini, L. Floreano, M. Stredansky, E. Vesselli, C. Hohner, M. Kettner, J. Libuda, C. M. Schneider, G. Zamborlini, M. Cinchetti, V. Feyer, *Small* **2021**, *17*, 2104779.

[22] G. Zamborlini, D. Lüftner, Z. Feng, B. Kollmann, P. Puschnig, C. Dri, M. Panighel, G. Di Santo, A. Goldoni, G. Comelli, M. Jugovac, V. Feyer, C. M. Schneider, *Nat. Commun.* **2017**, *8*, 335.

[23] H. M. Sturmeit, I. Cojocariu, M. Jugovac, A. Cossaro, A. Verdini, L. Floreano, A. Sala, G. Comelli, S. Moro, M. Stredansky, M. Corva, E. Vesselli, P. Puschnig, C. M. Schneider, V. Feyer, G. Zamborlini, M. Cinchetti, *J. Mater. Chem. C* **2020**, *8*, 8876–8886.

[24] K. A. Nguyen, M. S. Gordon, J. A. J. Montgomery, H. H. Michels, *J. Phys. Chem.* **1994**, *98*, 10072–10078.

[25] A. Snis, I. Panas, *Chem. Phys.* **1997**, *221*, 1–10.

[26] H. A. Duarte, E. Proynov, D. R. Salahub, *J. Chem. Phys.* **1998**, *109*, 26–35.

[27] F. Fuster, C. Dézarnaud-Dandine, H. Chevreau, A. Sevin, *Phys. Chem. Chem. Phys.* **2004**, *6*, 3228–3234.

[28] N. Taguchi, Y. Mochizuki, T. Ishikawa, K. Tanaka, *Chem. Phys. Lett.* **2008**, *451*, 31–36.
